# BCG therapy downregulates HLA-I on malignant cells to subvert antitumor immune responses in bladder cancer

**DOI:** 10.1172/JCI145666

**Published:** 2022-06-15

**Authors:** Mathieu Rouanne, Julien Adam, Camélia Radulescu, Diane Letourneur, Delphine Bredel, Séverine Mouraud, Anne-Gaëlle Goubet, Marion Leduc, Noah Chen, Tuan Zea Tan, Nicolas Signolle, Amélie Bigorgne, Michael Dussiot, Lambros Tselikas, Sandrine Susini, François-Xavier Danlos, Anna K. Schneider, Roman Chabanon, Sophie Vacher, Ivan Bièche, Thierry Lebret, Yves Allory, Jean-Charles Soria, Nicholas Arpaia, Guido Kroemer, Oliver Kepp, Jean Paul Thiery, Laurence Zitvogel, Aurélien Marabelle

**Affiliations:** 1 INSERM U1015, Gustave Roussy, Université Paris-Saclay, Villejuif, France.; 2 Department of Microbiology and Immunology, Vagelos College of Physicians and Surgeons, Columbia University, New York, New York, USA.; 3 Département d’Urologie, Hôpital Foch, UVSQ – Université Paris-Saclay, Suresnes, France.; 4 Département de Biologie et Pathologie Médicales, Gustave Roussy, Université Paris-Saclay, Villejuif, France.; 5 INSERM U1186, Gustave Roussy, Villejuif, France.; 6 Département de Pathologie, Hôpital Foch, UVSQ – Université Paris-Saclay, Suresnes, France.; 7 Master de Biologie, École Normale Supérieure de Lyon, Université Claude Bernard Lyon I, Université de Lyon, Lyon, France.; 8 Metabolomics and Cell Biology Platforms, Gustave Roussy Cancer Center, and; 9 Gustave Roussy, Université Paris-Saclay, Villejuif, France.; 10 Genomics and Data Analytics Core (GeDaC), Cancer Science Institute of Singapore, National University of Singapore, Singapore.; 11 INSERM U981, Gustave Roussy, Villejuif, France.; 12 INSERM U1163, Institut Imagine, Université de Paris, Paris, France.; 13 Département d’Innovation Thérapeutique et d’Essais Précoces (DITEP), Gustave Roussy, Université Paris-Saclay, Villejuif, France.; 14 ATIP-Avenir Group, INSERM U981, Gustave Roussy, Université Paris-Saclay, Villejuif, France.; 15 The CRUK Gene Function Laboratory and Breast Cancer Now Toby Robins Breast Cancer Research Centre, The Institute of Cancer Research, London, United Kingdom.; 16 Service de Génétique, Institut Curie, PSL Research University, Paris, France.; 17 Départment de Pathologie, Institut Curie, Saint-Cloud, France.; 18 CNRS UMR144, Paris, France.; 19 Herbert Irving Comprehensive Cancer Center, Columbia University, New York, New York, USA.; 20 Centre de Recherche des Cordeliers, Université de Paris, Sorbonne Université, INSERM U1138, Institut Universitaire de France, Paris, France.; 21 Pôle de Biologie, Hôpital Européen Georges Pompidou, AP-HP, Paris, France.; 22 Guangzhou Laboratory, Guangzhou, China.; 23 Centre d’Investigation Clinique de Biothérapies du Cancer (CICBT), Villejuif, France.

**Keywords:** Immunology, Oncology, Bacterial infections, Cancer, MHC class 1

## Abstract

Patients with high-risk, nonmuscle-invasive bladder cancer (NMIBC) frequently relapse after standard intravesical bacillus Calmette-Guérin (BCG) therapy and may have a dismal outcome. The mechanisms of resistance to such immunotherapy remain poorly understood. Here, using cancer cell lines, freshly resected human bladder tumors, and samples from cohorts of patients with bladder cancer before and after BCG therapy, we demonstrate 2 distinct patterns of immune subversion upon BCG relapse. In the first pattern, intracellular BCG infection of cancer cells induced a posttranscriptional downregulation of HLA-I membrane expression via inhibition of autophagy flux. Patients with HLA-I–deficient cancer cells following BCG therapy had a myeloid immunosuppressive tumor microenvironment (TME) with epithelial-mesenchymal transition (EMT) characteristics and dismal outcomes. Conversely, patients with HLA-I–proficient cancer cells after BCG therapy presented with CD8^+^ T cell tumor infiltrates, upregulation of inflammatory cytokines, and immune checkpoint–inhibitory molecules. The latter patients had a very favorable outcome. We surmise that HLA-I expression in bladder cancers at relapse following BCG does not result from immunoediting but rather from an immune subversion process directly induced by BCG on cancer cells, which predicts a dismal prognosis. HLA-I scoring of cancer cells by IHC staining can be easily implemented by pathologists in routine practice to stratify future treatment strategies for patients with urothelial cancer.

## Graphical abstract

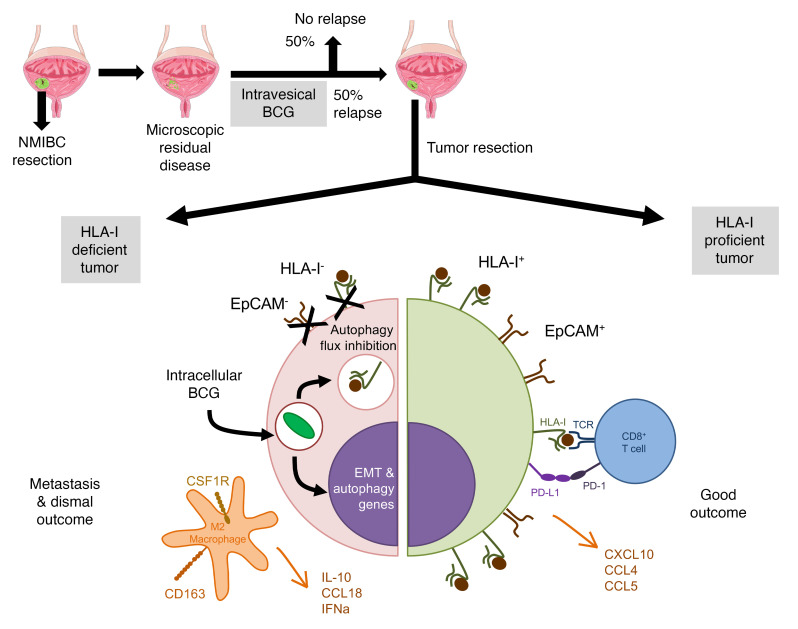

## Introduction

Bladder cancer is a heterogeneous disease that displays invasive and noninvasive histological features of the urothelium, as well as a wide spectrum of molecular alterations and subtypes (1, 2). Treatment of noninvasive tumors with high-risk features (carcinoma in situ [CIS], high-grade Ta [cancer cells involving the urothelium, the innermost layer of the bladder mucosa], any T1 [cancer cells involving the lamina propria, the connective tissue beneath the urothelium]) includes transurethral resection of the tumor, followed by intravesical instillations of bacillus Calmette-Guérin (BCG) (3). The origin of BCG traces back to the determined and perseverant work of Albert Calmette and Camille Guérin, who developed an attenuated form of live *Mycobacterium bovis* after 13 years of culturing and 231 passages (4). Today, BCG is still the only available vaccine against tuberculosis and one of the most successful cancer immunotherapies used as a standard adjuvant therapy for high-risk, nonmuscle-invasive bladder cancer (NMIBC). Despite a multitude of evidence for antitumor efficacy, half of the patients with high-risk NMIBC eventually develop tumor recurrence, and up to 30% of these patients will progress to secondary muscle-invasive bladder cancer (MIBC). Ultimately, 10% to 15% of patients die of metastatic disease (5, 6). Patients with secondary MIBC following BCG immunotherapy have worse oncological outcomes as compared with those with de novo primary MIBC. Additionally, such patients would not benefit from current standard cisplatin-based neoadjuvant chemotherapy, which suggests that tumor escape from intravesical BCG immunotherapy may select clones that are resistant to subsequent systemic chemotherapy (7).

Although recent studies have identified potential immune parameters that could affect the clinical response, the mechanisms of tumor resistance to BCG immunotherapy remain poorly understood (8–13). Indeed, modeling early-stage bladder cancer in mice is particularly challenging because of the rapid tumor growth inside the murine bladder wall (14). Additionally, tumor heterogeneity and the plasticity of urothelial cancer cells undermine our attempts to decipher the dynamics of immune escape under selective pressure. How urothelial cancer cells evade the antitumor immune response and whether such urothelial cancer cells could acquire extrinsic or intrinsic immune escape mechanisms upon BCG exposure remain unknown. Altogether, this highlights the crucial need for a more thorough understanding of the mechanisms of resistance to BCG immunotherapy to better stratify bladder cancer treatment strategies and identify new targetable pathways.

In this study, we investigated the effects of BCG immunotherapy on the biology of bladder cancers. We identified 2 distinct patterns of immune resistance after BCG exposure and propose a biologically relevant and clinically feasible stratification strategy for the treatment of patients with tumors progressing after intravesical BCG immunotherapy.

## Results

### Ex vivo BCG stimulation of fresh human bladder tumors and cancer cell lines induces HLA-I downregulation on cancer cells.

**Figure 1:**
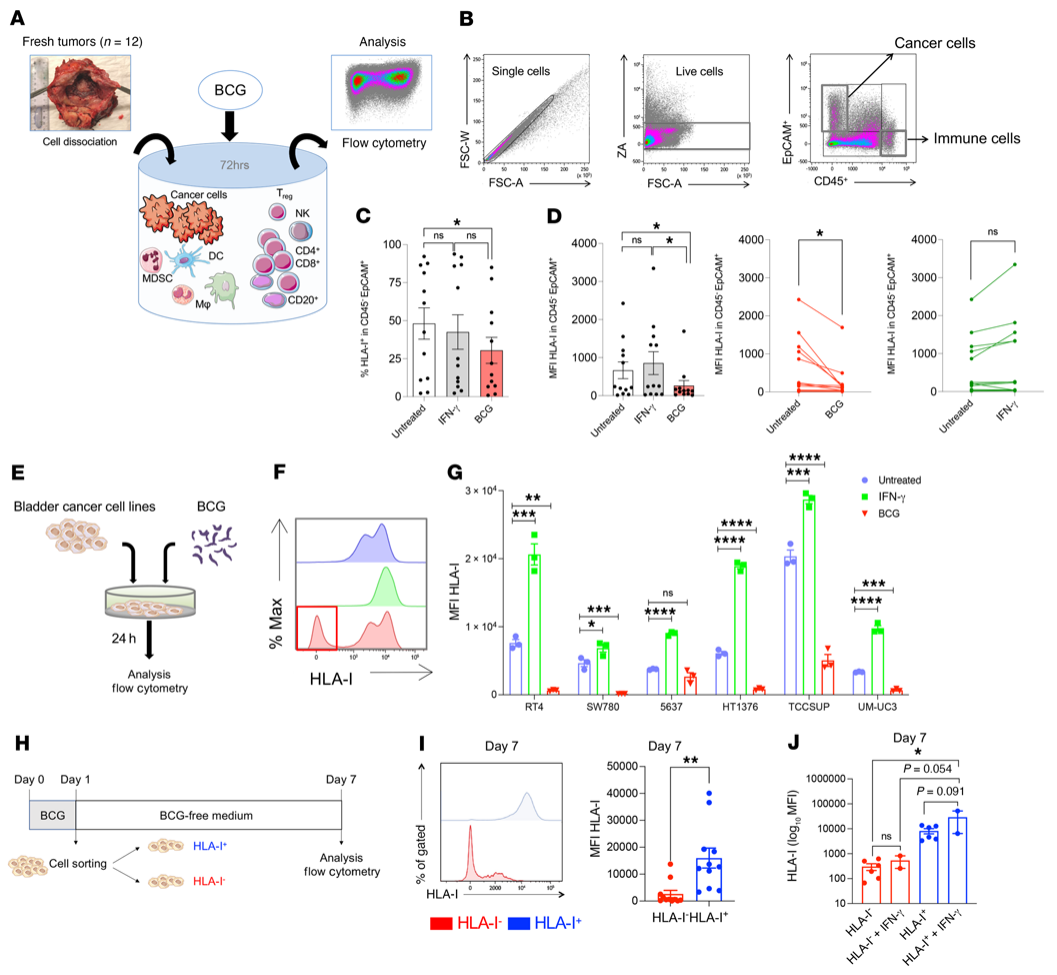
BCG induces HLA-I downregulation on cancer cells. (**A**) Cell suspensions from freshly dissociated human bladder tumors were cultured in triplicate in complete medium (untreated), stimulated with IFN-γ, or coincubated with BCG. (**B**) Gating strategy adopted to detect CD45^+^ immune cells and CD45^–^EpCAM^+^ cancer cells by flow cytometric analysis. (**C**) Proportions of HLA-I^+^CD45^–^EpCAM^+^ cells among live tumor cells following ex vivo BCG or IFN-γ stimulation of fresh human tumors (*n =* 12; 1-way ANOVA with Tukey’s post test). (**D**) MFI of HLA-I on live CD45^–^EpCAM^+^ cancer cells upon ex vivo BCG or IFN-γ exposure (*n =* 12 paired samples; 1-way ANOVA with Tukey’s post test). (**E**) High-grade (*n =* 4) and low-grade (*n =* 2) human bladder cancer cell lines were cultured in vitro in triplicate under 3 conditions: untreated, stimulated with IFN-γ, or coincubated with BCG. (**F**) Histogram showing HLA-I downregulation in a subset of cancer cells upon BCG exposure. (**G**) HLA-I MFI by flow cytometry 24 hours after in vitro BCG or IFN-γ exposure (*n =* 3 conditions per cell line; 1-way ANOVA with Tukey’s post test). (**H**) HLA-I^+^ and HLA-I^–^ cancer cells were sorted after 24 hours of coincubation with BCG. HLA-I^+^ and HLA-I^–^ cancer cells were cultured in BCG-free medium for 6 additional days, followed by flow cytometric analysis on day 7 to measure HLA-I expression on the cancer cells. (**I**) Sustained HLA-I MFI of cancer cells after 6 days in BCG-free medium. Left inset: Illustrative MFI from HLA-I^+^ (blue) and HLA-I^–^ (red). Graph shows cumulative data points of HLA-I MFI from 5 distinct cancer cells lines (*n =* 5 independent experiments using RT4, 5637, HT1376, TCCSUP, and UM-UC3 cancer cell lines; unpaired, 2-tailed t test). (**J**) HLA-I^+^ and HLA-I^–^ cancer cells were restimulated with IFN-γ for 24 hours after 3 days of culturing in BCG-free medium (1-way ANOVA with Tukey’s post test). All data are presented as the mean ± SEM. *P < 0.05, **P < 0.005, ***P < 0.001, and ****P < 0.0001.

The immune escape mechanisms of cancer cells following intravesical BCG remain poorly understood in patients with bladder cancer. To assess the effects of BCG on tumor-derived bladder cancer cells, we coincubated therapeutic BCG for 72 hours with whole-cell suspensions generated from mechanically and enzymatically dissociated fresh human bladder tumors (Figure 1A). All the samples were obtained from patients with clinically localized bladder tumors who were undergoing curative intent surgery (*n =* 12; Supplemental Table 1; supplemental material available online with this article; https://doi.org/10.1172/JCI145666DS1). By flow cytometry, we could then assess separately the proportions and effects of BCG on CD45^–^EpCAM^+^ bladder cancer cells and CD45^+^EpCAM^–^ WBCs from the same tumors (Figure 1B and Supplemental Figure 1). Although we expected to see most of the biological effects of BCG to occur on the WBC compartment, we found that coincubation with BCG induced a significant drop in the proportion of CD45^–^EpCAM^+^ cells expressing membrane HLA-I (Figure 1C). This diminution in the proportions of HLA-I^–^ cells was also associated with a significant diminution of HLA-I MFI, which reflects the total number of HLA-I molecules expressed on the outer membrane of cells (Figure 1D, left panel). This BCG effect on HLA-I expression was more dramatic in some tumors than in others (Figure 1D, middle and right panels) and was not found to be significant on WBCs (Supplemental Figure 1B). To explore the ability of BCG to activate lymphoid and myeloid cells in situ, we assessed the phenotype of CD45^+^ immune cells 72 hours after coculturing with BCG. We observed a drastic diminution of CD45^+^ immune cells 3 days after BCG exposure (Supplemental Figure 1C). However, we observed no variation among the immune cell subsets within the CD45^+^ cells. Therefore, the absolute quantity of immune cells after 72 hours of BCG exposure was diminished, but the relative proportion of lymphoid CD3^+^ T cells, CD20^+^ B cells, CD56^+^ NK cells, and CD11b^+^ myeloid cell subsets among live CD45^+^ cells remained stable in the BCG condition compared with the medium and IFN-γ controls (Supplemental Figure 1C). Additionally, the relative proportions of CD4^+^ and CD8^+^ T cells among live CD3^+^ T cells, together with FoxP3^+^ cells among live CD4^+^ T cells were also stable (Supplemental Figure 1C). Next, we evaluated the activation of immune cells by looking at the relative proportions of HLA-DR^+^ cells in CD11b^+^ and CD69^+^ cells and programmed cell death 1–positive (PD-1^+^) cells in CD3^+^ T cells. We observed no difference across conditions (Supplemental Figure 1D). Overall, we found that BCG did not induce activation markers on the surface of lymphoid or myeloid cells in the early-stage bladder tumors. However, BCG could induce a marked diminution of HLA-I molecules expressed on the surface of cancer cells.

### BCG induces a downregulation of HLA class I molecules on some cancer cells.

To further investigate the effect of BCG on HLA-I expression by cancer cells, we exposed in vitro low-grade (RT4, SW780) and high-grade (5637, HT1376, TCCSUP, and UM-UC3) human bladder cancer cell lines to therapeutic BCG (Figure 1E). We confirmed that HLA-I deficiency occurred in a subset of cancer cells in all of the 6 cell lines tested (Figure 1F and Supplemental Figure 1E). Similarly to the ex vivo data, we observed a concomitant, significant decrease in MFI for both HLA-I molecules (HLA-A/-B/-C subsets) and β-2 microglobulin (β2m) in high-and low-grade cancer cells 24 hours after BCG coincubation (Figure 1G and Supplemental Figure 1, F and G). Altogether, we confirmed that BCG induced HLA-I downregulation in a subset of cancer cells early (24 hours) after BCG exposure, independently of the presence of the immune and stromal cell compartments. We next evaluated whether HLA-I downregulation was sustained over time. To test this approach, we sorted cancer cells on the basis of HLA-I expression 24 hours after BCG exposure. Subsequently, we cultured HLA-I^+^ and HLA-I^–^ cells independently in BCG-free medium for 6 days (Figure 1H) and then analyzed HLA-I expression by flow cytometry on day 7. We still observed a significant reduction in HLA-I MFI in HLA-I^–^ cells (Figure 1I), which was not reversible by IFN-γ exposure (Figure 1J). Collectively, these data demonstrate that BCG may shape the immunogenicity of cancer cells, independently of the tumor immune microenvironment, by downregulating their membrane expression of HLA-I and β2m antigen-presenting molecules.

### BCG induces EMT characteristics in the subset of HLA-I^–^ cancer cells.

**Figure 2:**
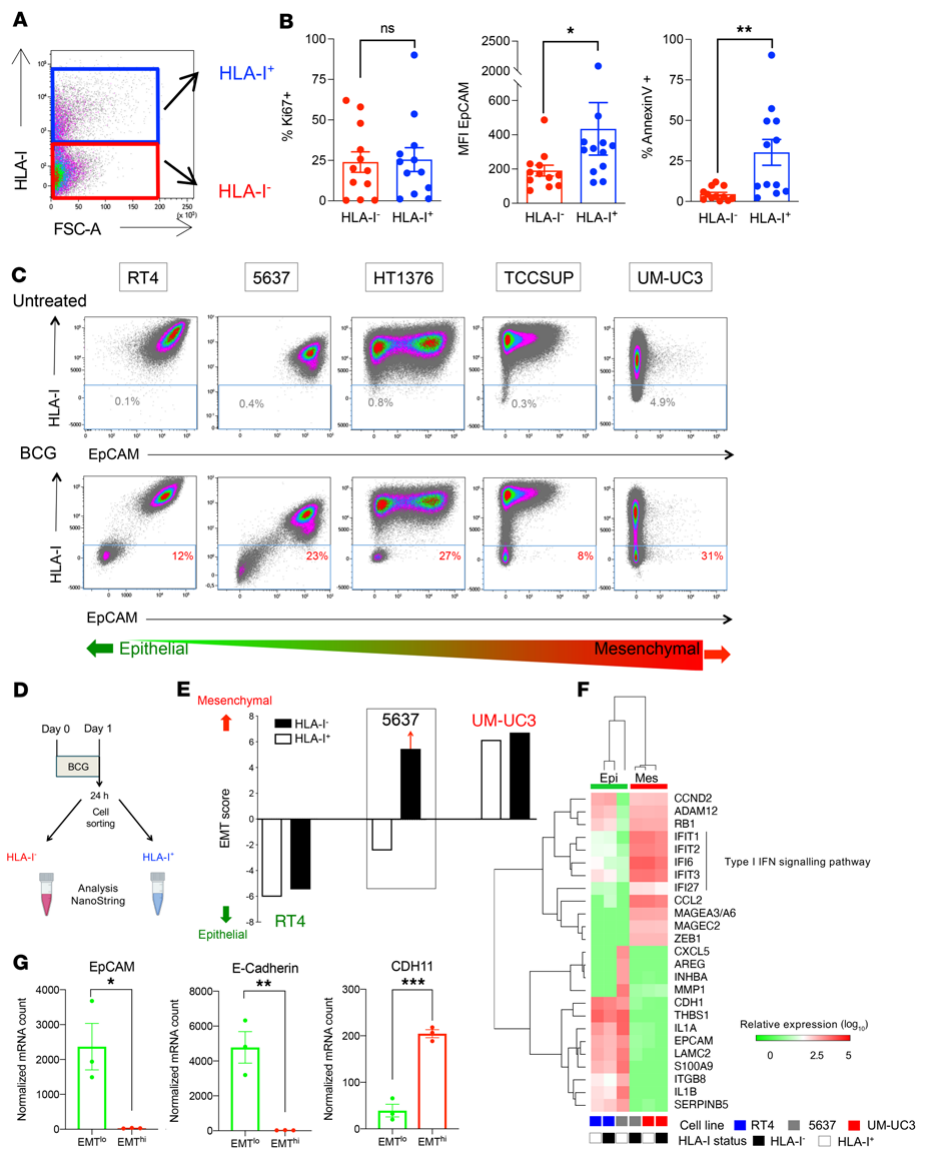
BCG induces EMT characteristics in the subset of HLA-I^–^ cancer cells. (**A**)Flow cytometric dot plot showing HLA-I^+^ and HLA-I^–^ cancer cells among live cells following ex vivo BCG stimulation of fresh human tumors for 72 hours. (**B**) MFI of EpCAM (*n =* 12; 1-way ANOVA with Tukey’s post test) and relative proportions of proliferative Ki67^+^ and apoptotic annexin-V^+^ cells (*n =* 12; paired, 2-tailed *t* test) among live cancer cells by flow cytometry. (**C**) Flow cytometric dot plots for HLA-I and EpCAM expression in live cancer cells. Dot plots illustrate the control condition at the top and the BCG condition at the bottom. Cancer cells were ordered according to their baseline expression of EpCAM (MFI), ranging from the left (epithelial type, RT4) to the right (mesenchymal type, UM-UC3). (**D**) Cancer cells (RT4, 5637, and UM-UC3) were coincubated with BCG for 24 hours and sorted on the basis of their HLA-I membrane expression. Total RNA extraction of HLA-I^+^ and HLA-I^–^ cancer cells was performed. (**E**) The EMT score based on NanoString IO360 transcriptomic data was obtained for HLA-I^+^ and HLA-I^–^ cancer cells. HLA-I^–^ 5637 cancer cells showed the strongest shift toward a mesenchymal score. (**F**) Unsupervised hierarchical clustering analysis of cancer cells according to EMT status, depicted per cell line (columns), HLA-I status (columns), and genes expressed (rows). (**G**) Epithelial (*CDH1* [E-cadherin] and *EPCAM*) and mesenchymal (*CDH11*) absolute mRNA expression after BCG (red, EMT^hi^ UM-UC3 and 5637 HLA-I^–^; green, EMT^lo^ RT4 and 5637 HLA-I^+^). Unpaired, 2-tailed *t* test. All data are presented as the mean ± SEM. *P < 0.05, **P < 0.005, ***P < 0.001.

Next, we investigated whether cancer cells with BCG-induced HLA-I downregulation (HLA-I^–^) have specific characteristics in comparison with HLA-I^+^ cancer cells upon ex vivo exposure of fresh human bladder tumors to BCG (Figure 2A). First, we assessed the proliferative activity and apoptosis of cancer cells according to their HLA-I membrane expression as assessed by flow cytometry. Interestingly, the percentage of Ki67^+^ cells did not differ according to HLA-I expression (Figure 2B). However, we observed a significant diminution of epithelial cellular adhesion molecule (EpCAM) membrane expression by MFI and a lower proportion of annexin-V^+^ cells within HLA-I^–^ cancer cells (Figure 2B, middle and right panels, respectively). Together, these data suggested that BCG-induced HLA-I downregulation could be associated with apoptosis resistance and acquisition of mesenchymal characteristics in cancer cells.

To further evaluate the link between HLA-I and EpCAM expression, we measured the membranous coexpression of HLA-I and EpCAM by flow cytometry on a broad range of bladder cancer cell lines, ranging from an epithelial to a mesenchymal phenotype as previously described (Supplemental Figure 2D and ref. 15). We observed an association between HLA-I downregulation and EpCAM loss in epithelial cells (Figure 2C). Additionally, HLA-I^–^EpCAM^–^ cells were characterized by lower forward scatter (FSC-A) and side scatter (SSC-A), cell shrinkage, flattened bodies, and loss of intercellular adhesion in contrast to the parental cell line (Supplemental Figure 2, A and B). As seen with HLA-I downregulation, EpCAM loss was maintained over time (Supplemental Figure 2C). Importantly, these data suggest that BCG could induce an epithelial-mesenchymal transition (EMT) in a subset of cancer cells.

To better explore this hypothesis, we analyzed the EMT status of cancer cells 24 hours after BCG coincubation. In this experimental study, we used 3 cell lines with the most extreme EMT status (as previously defined by a quantification algorithm [ref. 15] and as outlined in Supplemental Figure 2D), including the epithelial cell line RT4, the mesenchymal cell line UM-UC3, and the 5637 cancer cell line with intermediate EMT status. To identify a shift toward a mesenchymal state, we sorted the HLA-I^+^ and HLA-I^–^ cancer cells 24 hours after BCG exposure and extracted RNA from these 2 cell subsets (Figure 2D). We next calculated the EMT score of HLA-I^+^ and HLA-I^–^ cells for each cancer cell line on the basis of a previously identified epithelial and mesenchymal gene expression data set (Supplemental Table 2). Although no difference was observed between HLA-I^+^ and HLA-I^–^ cells for RT4 and UM-UC3 cell lines, we identified a substantial shift toward a mesenchymal score in HLA-I^–^ 5637 cancer cells (Figure 2E).

To further understand whether BCG exposure induces transcriptional modifications of cancer cells, we identified the top 25 differentially expressed genes between EMT^lo^ and EMT^hi^ cell lines among a predefined NanoString panel of 770 gene products involved in cancer-immune crosstalk. Next, we applied unsupervised hierarchical analyses to RT4, 5637, and UM-UC3 on the basis of these 25 top genes quantified after BCG exposure. We confirmed that EMT^lo^ 5637 cells clustered with the epithelial RT4 cell line and EMT^hi^ 5637 cells with the mesenchymal UM-UC3 cell line (Figure 2F). Notably, HLA-I^–^ cancer cells acquiring a mesenchymal status were associated with enhanced mRNA levels of the IFN-stimulated genes *IFIT1*, *IFIT2*, *IFIT3*, *IFI6*, *IFI27*, and *CCL2*. We also noticed increased expression of the EMT activator *ZEB1*, the tumor-associated antigens *MAGEA3/A6* and *MAGEC2*, and metalloproteinase in EMT^hi^ cancer cells (Figure 2F). By contrast, HLA-I^+^EMT^lo^ cancer cells were associated with enhanced *IL1A*, *IL1B*, and *CXCL5* mRNA expression. We also noticed higher levels of *EPCAM* and *CDH1* (E-cadherin) mRNA in this epithelial group, as well as significantly lower levels of *CDH11* mRNA compared with levels in the mesenchymal group (Figure 2G).

Overall, these data suggest that BCG exposure may induce a mesenchymal transition associated with HLA-I and EpCAM downregulation in a subset of epithelial cancer cells. Importantly, HLA-I downregulation was associated with a mesenchymal state in a subset of cancer cells, which displayed a type I IFN pathway, in contrast to the proinflammatory IL-1 cytokine pathway observed in cells with an epithelial transcriptomic phenotype. Of note, we tried to recapitulate the BCG-induced HLA-I downregulation findings in the MB49 and UPPL1541 transplantable syngeneic murine models of bladder cancer but realized that these murine cancer cell lines were already HLA-I^–^ (Supplemental Figure 2E, upper and lower panels respectively).

### BCG induces greater inflammatory responses in HLA-I^+^ cancer cells than in HLA-I^–^ cells, and these responses are further enhanced upon BCG reexposure.

**Figure 3:**
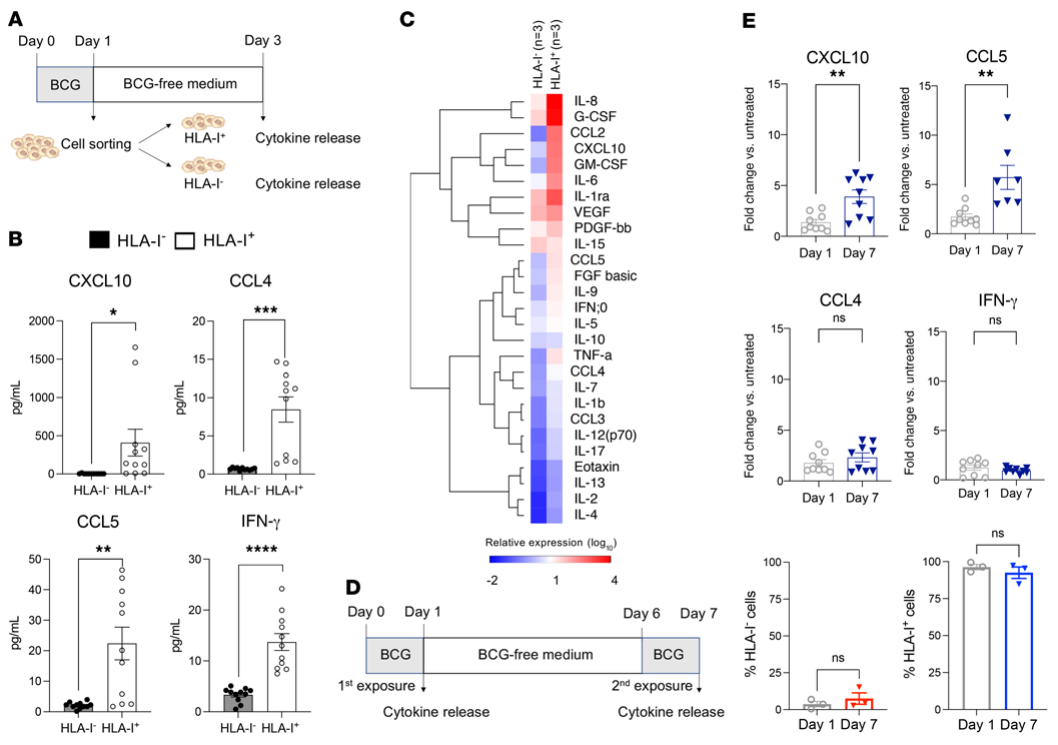
BCG induces greater inflammatory responses in HLA-I^+^ than in HLA-I^–^ cancer cells, and these responses are further enhanced upon BCG reexposure. (**A**) Cytokine and chemokine release in the supernatant was measured separately in HLA-I^+^ and HLA-I^–^ cancer cells 24 hours after coincubation with BCG and independent culturing in BCG-free medium for 3 days (*n =* 3 independent experiments in triplicate per cell line). (**B**) HLA-I^+^ cancer cells released significant levels of CXCL10, CCL4, CCL5, and IFN-γ upon BCG exposure, as opposed to HLA-I^–^ cancer cells, which released low levels of cytokines. (**C**) Heatmap showing cytokine and chemokine relative expression in HLA-I^+^ and HLA-I^–^ cancer cells 24 hours after coincubation with BCG. (**D**) BCG restimulation assay evaluating cytokine and chemokine recall responses of cancer cells (5637 cell line). The first stimulation was for 24 hours, followed by 5 days of culturing in BCG-free medium and a second BCG exposure for 24 hours. Cytokine and chemokine production was measured after the first and the second stimulations. (**E**) Cancer cells initially primed with BCG displayed significantly increased levels of CXCL10 and CCL5 upon restimulation with BCG 7 days later (upper panels), but this secondary exposure to BCG did not modify the secretion levels of CCL4 or IFN-γ (middle panels) or the proportions of cancer cells expressing HLA-I (lower panels). *P < 0.05, **P < 0.005, ***P < 0.001, and ****P < 0.0001, by unpaired, 2-tailed Student’s *t* test.

To better characterize the biology of cancer cells according to their HLA-I status after BCG exposure, we investigated the levels of cytokines and chemokines released by cancer cells 24 hours after BCG exposure. Bladder cancer cells were sorted on the basis HLA-I expression after 24 hours of exposure to BCG and were cultured independently in BCG medium–free environment for an additional 72 hours (Figure 3A). Supernatants were then titrated for their cytokine and chemokine content. We found strong differences in the secretory profile of HLA-I^+^ and HLA-I^–^ cancer cells (Figure 3, B and C, and Supplemental Figure 3). Importantly, HLA-I^+^ cancer cells secreted sustained levels of the chemotactic factors CXCL10, CCL4, CCL5, and IFN-γ cytokine in contrast to the HLA-I^–^ cancer cells (Figure 3B). Overall, these results demonstrated that the release of T cell chemoattractants was high in HLA-I^+^ cancer cells upon BCG exposure but very low in HLA-I^–^ cancer cells.

To test whether the restimulation of cancer cells with BCG would enhance their cytokine production, we coincubated cancer cells with BCG for 24 hours. Then, cells were cultured in BCG-free medium for 5 days. On day 6, the cancer cells were restimulated with BCG for 24 hours (Figure 3D). We observed dramatic increases in the levels of CXCL10 and CCL5 release upon a second stimulation with BCG, although the proportion of HLA-I^+^ and HLA-I^–^ cells remained unchanged (Figure 3E). Overall, these data provide evidence that the cancer cells primed with BCG displayed a stronger recall response upon BCG reexposure.

### BCG-infected cancer cells downregulate HLA class I and EpCAM membrane expression.

We explored the mechanism by which BCG induced HLA-I downregulation on cancer cells. BCG is known to activate TLR2, TLR4, and TLR9 (16). Therefore, we investigated whether HLA-I downregulation was directly dependent on whole, live bacteria or if it could be induced directly by the activation of TLR2, TLR4, and TLR9. To address this question, we compared the effects of live attenuated strains of BCG with synthetic agonists of TLR2, TLR4, and TLR9. Of note, neither TLR agonists alone nor in combination could induce HLA-I downregulation on cancer cells (Supplemental Figure 4A). These data suggest that live bacteria rather than TLR stimulation are required to induce HLA-I downregulation in cancer cells.

**Figure 4:**
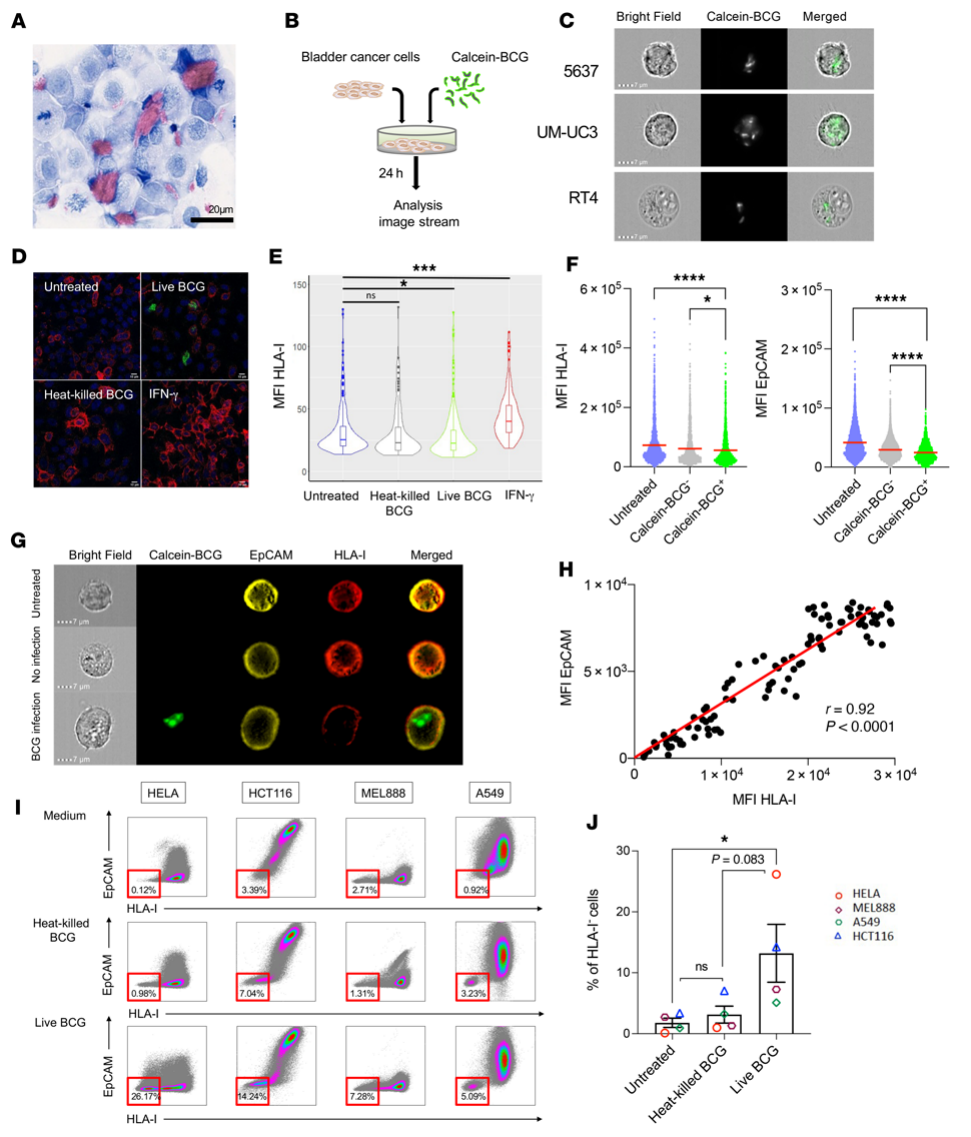
BCG-infected cancer cells downregulate HLA-I and EpCAM membrane protein expression. (**A**) Ziehl-Neelsen staining for UM-UC3 cancer cells coincubated with BCG (MOI of 10) for 24 hours showed isolated bacteria inside cancer cells and agglomerates of BCG outside of cancer cells. Scale bar: 20 μm. (**B**) Experimental setting to evaluate the coincubation of cancer cells (RT4, 5637, UM-UC3) with calcein-labeled BCG. (**C**) Intracytoplasmic visualization of calcein-labeled BCG inside the cytoplasm of cancer cells. Original magnification, ×60. (**D**) Immunofluorescence staining of HLA-I (red), and calcein (green) in human bladder cancer cells following in vitro exposure to calcein-labeled BCG, heat-killed, calcein-labeled BCG, or IFN-γ for 24 hours. Original magnification, ×63. (**E**) HLA-I MFI quantification per confocal image analysis on cancer cells cocultured with heat-killed or live BCG. HLA-I MFI data is given for every single cell acquired for untreated (*n* = 244 cells), BCG (*n* = 239), heat-killed BCG (*n* = 160), and IFN-γ (*n* = 175). Only live BCG induced a significant downregulation of HLA-I expression on cancer cells (1-way ANOVA with Tukey’s post test). (**F**) MFI for HLA-I and EpCAM in BCG infected (intracellular calcein-BCG^+^) versus noninfected (calcein-BCG^–^) cancer cells. Controls were untreated cancer cells (1-way ANOVA with Tukey’s post test). (**G**) Representative images of BCG-untreated, BCG-infected, and BCG-noninfected cancer cells 24 hours after BCG coincubation. Original magnification, ×60. (**H**) Spearman’s correlation for the MFI of HLA-I and EpCAM in 100 cancer cells, 24 hours after BCG coincubation. (**I**) HLA-I and EpCAM expression on melanoma (MEL888), lung (A549), colorectal (HCT116), and cervical (HeLa) carcinoma cells when cultured with control media, heat-killed BCG, or live BCG. Relative proportion of HLA-I^–^ cancer cells (percentage) among live cells (1-way ANOVA with Tukey’s post test). (**J**) Proportion of HLA-I^–^ cells upon 24 hours of coincubation with either heat-killed or live BCG on HELA, HCT116, MEL888, and A549 cancer cell lines (1-way ANOVA with Tukey’s post test). Data are presented as the mean ± SEM. *P < 0.05, ***P < 0.001, and ****P < 0.0001.

Because mycobacteria can reside within eukaryotic cells, we hypothesized that live bacteria from therapeutic BCG could infect cancer cells. First, we stained a coculture of bladder cancer cells and BCG with Ziehl-Neelsen dye and could detect some BCG bacteria within the cytoplasm of cancer cells (Figure 4A). Next, we used calcein to confirm the presence of live bacteria in the therapeutic BCG. Calcein is a fluorescent dye that can be transported through the cellular membrane into live organisms, which makes it useful for testing the viability of bacteria and tracking intracellular infection by live bacteria. To determine whether either live or dead BCG was required to observe the effect on cancer cell HLA-I downregulation, we first evaluated the best way —heat or UVB exposure — to eradicate viable (calcein^+^) BCG. We found that heat was the most efficient technique to kill BCG (Supplemental Figure 4C). To assess whether live bacteria from therapeutic BCG was able to enter cancer cells, we coincubated calcein-labeled BCG together with 3 bladder cancer cell lines (RT4, 5637, and UM-UC3; Figure 4B). As previously reported by Redelman-Sidi, we found that the susceptibility of cancer cells to infection by BCG was variable across the cell lines and was higher in the mesenchymal type of cancer cell lines (UM-UC3) than in the epithelial type (RT4), notably at a lower ratio of cancer cells/BCG (with a MOI as low as 10 bacilli/cancer cell) (Figure 4C and Supplemental Figure 4B). Then we performed a comparison of the direct effects of calcein-labeled live BCG versus heat-killed BCG on the expression of HLA-I on bladder cancer cells. By immunofluorescence staining for membrane HLA-I expression, we found that the intensity of HLA-I membrane expression was significantly lower when the cancer cells were exposed to live BCG but not to heat-killed BCG (Figure 4, D and E). Indeed, live BCG (calcein^+^) penetrated the cytoplasm of cancer cells and downregulated HLA-I expression (Figure 4D, bottom left image), whereas no live BCG was observed in cancer cells when calcein-labeled BCG was pretreated with heat (80°C for 60 minutes), and HLA-I expression was not downregulated.

Next, we used ImageStream technology to assess at the single-cell level the correlation between BCG internalization (calcein^+^ cells) and the intensity of membrane HLA-I expression by cancer cells. We confirmed that the cancer cells infected by BCG (calcein^+^ cancer cells) were the ones with significantly lower expression of membrane HLA-I (Figure 4F, left panel). We also found that the cancer cells infected by BCG expressed significantly lower membrane levels of EpCAM (Figure 4F, right panel). By image stream analysis providing single-cell data, we found a strong correlation between HLA-I and EpCAM MFIs (*r* = 0.92; *P <* 0.0001; Figure 4, G and H). We then tested other cancer cell lines from other histologies and confirmed that BCG could also induce a downregulation of their membrane HLA-I expression (Figure 4I), which was also specific to live BCG but not observed with heat-killed BCG (Figure 4J). Together, these data demonstrate that live, attenuated mycobacteria from clinical-grade BCG were able to directly infect a subset of human cancer cells and that this intracellular infection was associated with a significant diminution of HLA-I and EpCAM membrane expression.

### BCG-induced HLA-I downregulation is a posttranscriptional phenomenon associated with inhibition of autophagy flux.

**Figure 5:**
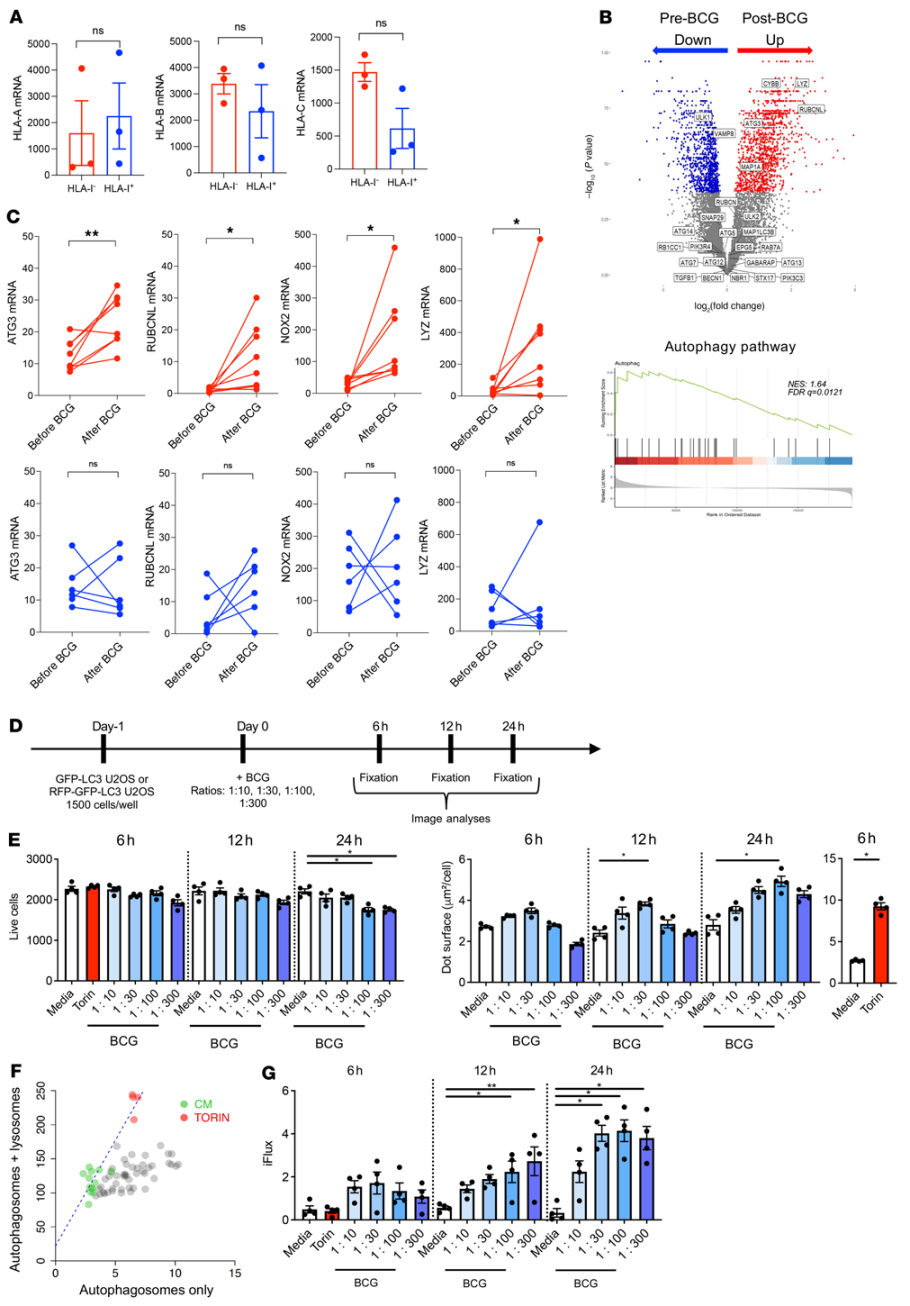
BCG induces posttranscriptional downregulation of HLA-I associated with inhibition of autophagy flux. (**A**) HLA-IA/-B/-C mRNA expression in HLA-I^–^ and HLA-I^+^ cancer cells following BCG exposure for 24 hours (unpaired, 2-tailed Student’s *t* test). (**B**) Gene expression in tumors that became HLA-I^–^ upon BCG therapy. Top: differentially expressed genes in paired human bladder tumors (*n =* 8; significantly up-/downregulated genes are identified by color dots; *P* < 0.05). Bottom: Gene set analysis showing enrichment of autophagy pathway genes (*n =* 8). (**C**) Before and after BCG therapy, paired analysis of autophagy-related gene expression levels in bladder tumors with decreasing (red) or increasing (blue) levels of HLA-I expression after BCG (paired, 2-tailed Student’s *t* test). (**D**) U2OS cells were engineered to express LC3 (autophagosome marker) and were fused to GFP with or without RFP. LC3-GFP cells, with or without RFP U2OS cells, were seeded overnight before adding BCG and cocultured for 6 hours, 12 hours, and 24 hours at different BCG MOI. Torin (1 μM; autophagy inducer) was used as a positive control and media as a negative control. (**E**) LC3-GFP U2OS cells were cultured for 6 hours, 12 hours, and 24 hours with BCG (MOI of 1:10, 1:30, 1:100, and 1:300), torin (1 μM), or media. Live cells counts (left) and GFP-LC3 puncta surface (right). Statistical analysis was determined by Kruskal-Wallis ANOVA and Dunn’s multiple-comparison test with media as the control. Data are from 1 of 2 independent experiments and are shown as the mean ± SEM of 4 technical replicates. (**F**) LC3-GFP-RFP U2OS cells were cocultured for 6 hours, 12 hours, and 24 hours with BCG (MOI of 1:10, 1:30, 1:100, and 1:300), torin (1 μM), or media. The linear regression curve is between the total surface of yellow (red plus green) and red (autophagosomes plus autophagolysosomes) dots. The negative control (media) is shown in green and the positive control (torin) in red. The gray dots indicate wells with BCG. (**G**) Quantification of the flux inhibition (iFlux) score. Kruskal-Wallis ANOVA and Dunn’s multiple-comparison test were performed, with media as the control. Data from 1 experiment are shown as the mean ± SEM of 4 technical replicates. *P < 0.05 and **P < 0.005.

To further understand the mechanism of BCG-induced downregulation of HLA-I, we tested the hypothesis that this effect could be related to lower expression of HLA-I genes. We performed NanoString analysis of 3 human bladder cancer cell lines after cell sorting based on their HLA-I status upon BCG exposure. This analysis revealed no significant differences in HLA-A, -B, or -C levels of gene expression in cells classified as HLA-I^+^ or HLA-I^–^ by protein expression (per flow cytometry pan–HLA-I staining) (Figure 5A, upper, middle, and lower panels, respectively). We also performed RNA-Seq of 14 paired human tumors before and after BCG (Supplemental Table 3) and assessed the genes that were differentially expressed in HLA-I–deficient tumors after BCG relapse (Figure 5B, upper panel). We found that several genes involved in the autophagy pathway had significant changes in expression levels (Figure 5B, lower panel, and Supplemental Table 4). More specifically, the autophagy-associated genes *ATG3*, *RUBCNL*, *NOX2*, and *LYZ* were significantly upregulated in the tumors with HLA-I downregulation after BCG relapse (as determined by IHC staining) (Figure 5C, upper panel). To better assess the relationship between BCG-induced downregulation of HLA-I and autophagy, we used the U2OS osteosarcoma LC3-GFP cell line, with or without the RFP reporter cell line (Supplemental Figure 5A). First, we confirmed that HLA-I downregulation was also induced upon exposure of therapeutic BCG to U2OS osteosarcoma cells (Supplemental Figure 5B). This phenomenon was BCG dose dependent and could not be overcome by concomitant IFN-γ exposure (Supplemental Figure 5, C and D). Then, we exposed U2OS LC3-GFP cells to increasing concentrations of BCG (MOI ranging from 1:10 to 1:300) (Figure 5D). We found that only very high doses beyond 24 hours of BCG exposure were cytotoxic to the U2OS cancer cells (Figure 5E, left panel). We also found that the total surface of the GFP dots (autophagosomes) per cell (μm^2^/cell) increased upon BCG treatment, without major changes in the dot counts. An increase in the surface area of the GFP dots without an increase in the number of dots can be linked to a phenomenon of inhibition of autophagic flux. This phenomenon is an inhibition of the degradation of autophagosomes, which can intervene at several levels: recruitment of lysosomes, fusion of lysosomes with autophagosomes, or degradation of autolysosomes. Autophagosomes then accumulate in the cytoplasm and aggregate. This question was subsequently tested on the LC3-GFP plus RFP tandem cell line, which allows one to track the generation of autophagolysosomes and study autophagy fluxes. We found an accumulation of autophagolysosomes in the cytoplasm of U2OS cancer cells in favor of an autophagy flux inhibition (Figure 5F). Inhibition of the autophagy flux was found to be BCG dose dependent after 12 hours of BCG coculturing with U2OS cells (Figure 5G). Altogether, we found that BCG-induced HLA-I downregulation was associated with inhibition of autophagy flux.

### Two distinct mechanisms of cancer cell immune escape steer the outcome of BCG immunotherapy.

**Figure 6:**
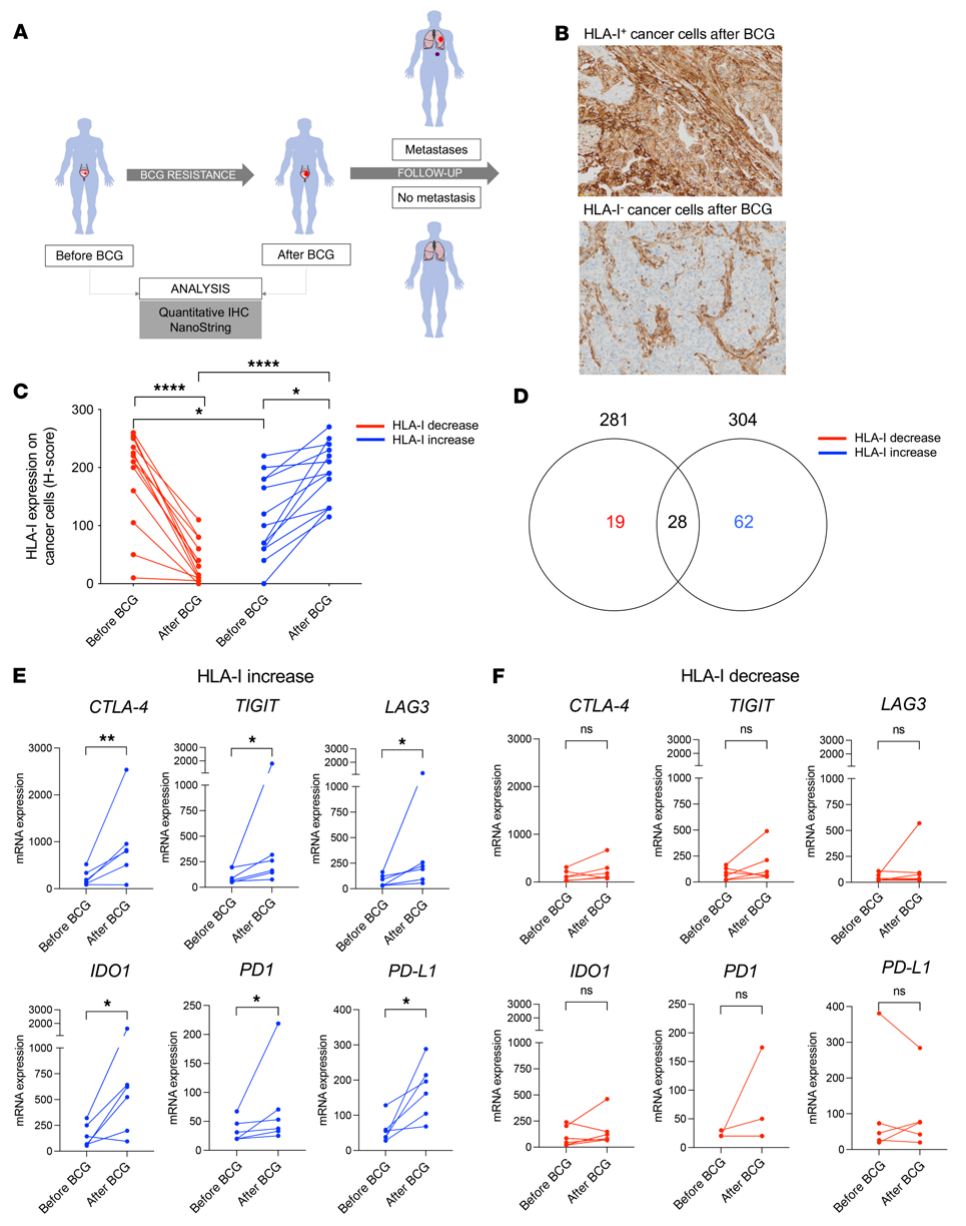
Cancer cell HLA-I downregulation upon BCG therapy is associated with an absence of T cell checkpoint modulation in the TME. (**A**) A longitudinal cohort of patients with BCG-resistant or refractory bladder cancer was identified (*n =* 27). Paired bladder tumors were evaluated before and after BCG immunotherapy–acquired resistance. (**B**) Representative images of HLA-I^+^ or HLA-I^–^ IHC stainings from bladder tumors with acquired resistance to BCG immunotherapy. Scale bar: 50 μm. (**C**) Relapsing/refractory bladder tumors displayed either downregulation (red) or upregulation (blue) of HLA-I upon BCG immunotherapy (1-way ANOVA with Tukey’s post test). (**D**) Venn diagram depicting the number of upregulated transcripts (*P <* 0.05) between paired tumors before and after BCG (*n =* 6) according to the evolution of HLA-I expression at the proteomic (i.e., IHC) level (left circle, number of upregulated genes in tumors with decreased HLA-I; right circle, number of upregulated genes in tumors with increased HLA-I). Only genes differentially upregulated (i.e., *P <* 0.05; log_2_ fold change ≥2) before and after BCG immunotherapy were selected from the NanoString analysis. (**E** and **F**) Paired representation of the absolute expression of immune checkpoint inhibitory receptor and ligand mRNAs before and after BCG relapse (red: HLA-I decrease, *n =* 6; blue: HLA-I increase, *n =* 6). Paired, 2-tailed Student’s *t* test. *P < 0.05, **P < 0.005, and ****P < 0.0001.

To determine whether HLA-I downregulation upon BCG therapy indeed occurred in vivo and affected the outcome of patients with bladder cancer, we studied a cohort of 27 patients who relapsed after BCG immunotherapy. These patients were first treated with intravesical BCG for high-risk NMIBC, which subsequently progressed to MIBC (Figure 6A and Supplemental Table 5). Longitudinal tumor samples obtained from paired primary and secondary bladder tumors before and after BCG were collected for analyses. To identify specific alterations of the immune microenvironment associated with acquired resistance to BCG, we performed in situ immune profiling using a predefined NanoString panel of 730 immune and inflammatory gene products and a 6-marker IHC panel (Supplemental Figure 6). We first measured the evolution of HLA-I expression on cancer cells before BCG therapy and upon relapse after BCG therapy in paired tumor samples (Figure 6B). Although there was no obvious difference between the pre-BCG and post-BCG relapse levels of HLA-I expression in tumors (Supplemental Figure 7A), upon paired analysis of the tumor samples, we detected a profound downregulation of HLA-I on cancer cells in half of the tumor cohort (*n =* 13 of 27) (Figure 6C). By contrast, HLA-I upregulation occurred in the second half (*n =* 14 of 27) of the cohort. Then, we hypothesized that the tumor’s HLA-I phenotype could affect the in situ inflammatory responses and subsequently orchestrate a specific tumor immune microenvironment. To test this hypothesis, we evaluated the differential gene expression profiles before BCG therapy and following BCG relapse after segregating the tumors according to whether HLA-I expression increased or decreased upon treatment (Figure 6D and Supplemental Figure 6).

**Figure 7:**
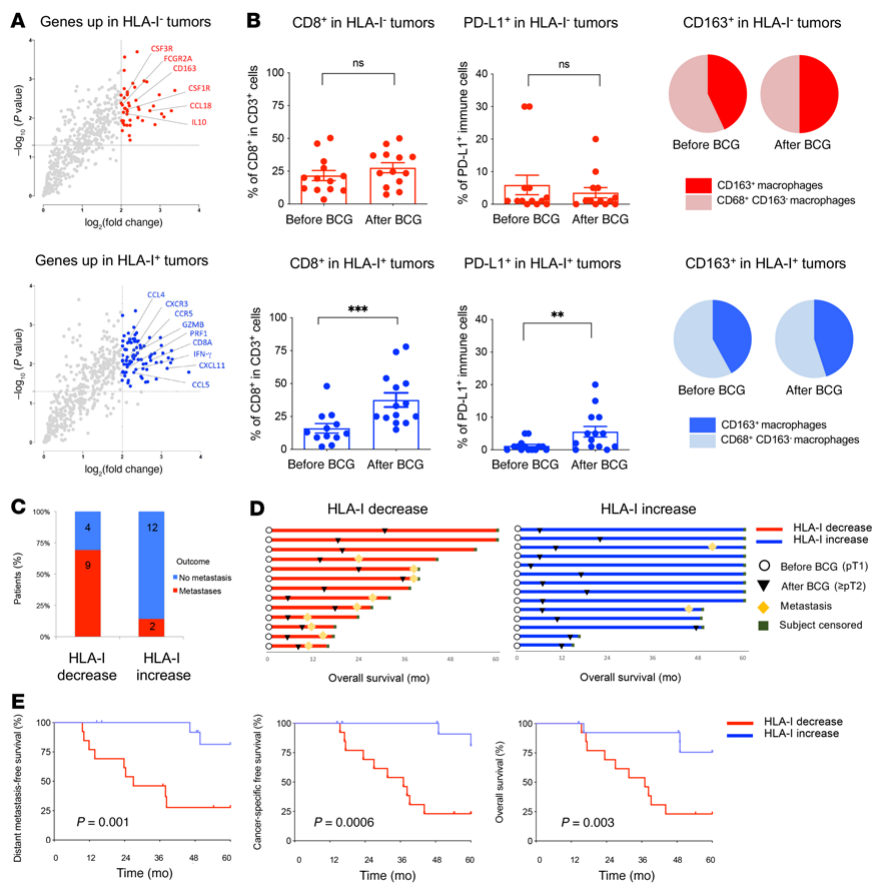
Two distinct mechanisms of cancer cell immune escape with opposite HLA-I dynamics steer the outcome of BCG immunotherapy. (**A**) Volcano plots showing genes that were upregulated in tumors with decreased HLA-I (HLA-I^–^ tumors; upper panel, in red) or in tumors with increased HLA-I (HLA-I^+^ tumors; lower panel, in blue), according to HLA-I expression at the protein level (per IHC) after BCG immunotherapy. Significantly upregulated genes of interest in each group are identified by colored dots (*P* < 0.05 and a log_2_ fold change ≥2). (**B**) IHC immune profiling of bladder tumor samples before and after BCG (*n =* 27). The density of CD8^+^ in CD3^+^ cells (percentage) and of PD-L1 expression on immune cells (percentage) is shown (paired, 2-tailed Student’s *t* test). Cumulative pie charts show the mean level of CD163^+^CD68^+^ cells among intratumoral macrophages (CD68^+^) before and after BCG (*n =* 27). Upper panel shows tumors with decreased HLA-I (HLA-I^–^ tumors; in red), and lower panel shows tumors with increased HLA-I (HLA-I^+^ tumors; in blue). **P < 0.005 and ***P < 0.00, by paired, 2-tailed Student’s *t* test. (**C**) Number and proportion of patients who developed distant metastasis during follow-up according to the evolution of HLA-I expression in their bladder tumors before and after BCG (red, HLA-I decrease; blue, HLA-I increase). (**D**) Swimmer plot depicting overall survival, the time to development of resistance to BCG, and the timing of distant metastasis for individual patients with bladder cancer according to the evolution of HLA-I expression in their tumor before and after BCG (red, HLA-I decrease; blue, HLA-I increase). (**E**) Distant metastasis–free survival, cancer-specific survival, and OS in the cohort of patients with bladder cancer (*n =* 27) according to the evolution of HLA-I expression in their tumors before and after BCG (red, HLA-I decrease; blue, HLA-I increase). *P* values were determined by log-rank test.

We identified a significant upregulation of genes expressed by T cells (CD8^+^, CD4^+^) and of their immune-checkpoint inhibitory receptors in relapsing tumors following BCG immunotherapy (Supplemental Figure 7B). However, looking separately at the transcriptome from tumors with either an up- or downregulation of HLA-I, we discovered that *CTLA4*, *TIGIT*, *LAG3*, programmed death ligand 1 (*PDL1*), *PD1*, and *IDO1* genes were selectively upregulated in tumors, which maintained or increased their HLA-I expression after BCG therapy (Figure 6E). In contrast, tumors that showed downregulation of HLA-I expression after BCG therapy did not have significantly modified levels of T cell immune checkpoint expression (Figure 6F). Additionally, we identified a distinct set of genes differentially expressed according to their tumor HLA-I subtypes (Figure 6D). Notably, we observed a “myeloid-suppressive” type of immune microenvironment with increased levels of CD163, CSF-1R, CSF-3R, and IL-10 expression in tumors expressing low levels of HLA-I following BCG relapse (Figure 7A, upper panel). In contrast, tumors with strong HLA-I^+^ expression were associated with a “T cell–suppressive” type of immune microenvironment characterized by increased levels of *CD8A*, *GZMB*, *PFR1*, and *IFNG* gene expression after BCG (Figure 7A, lower panel). Furthermore, we observed in this latter subgroup a significant upregulation of CXCL9/-10/-11, CCL4, and CCL5 chemokine expression, together with their specific receptors CXCR3 and CCR5 (Figure 7A, lower panel). Next, we evaluated the EMT score in serial tumor samples, starting from BCG-naive NMIBC to muscle-invasive progression and distant metastatic disease after BCG therapy. Similar to our in vitro data, we found that an increase in the EMT score correlated with downregulation of HLA-I expression over the progression of the disease (Supplemental Figure 7C).

To further validate our findings at the protein and cellular levels, we investigated the evolution of tumor-infiltrating lymphocytes (TILs) by IHC before and after BCG (Supplemental Figure 7D). Again, we could not find overall changes for those markers in tumors before BCG or after BCG relapse (Supplemental Figure 7E). However, when we looked at the data in a paired manner, we confirmed that CD8^+^ T cells and PD-L1 were significantly increased on immune cells in tumors expressing high levels of HLA-I at relapse following BCG (Figure 7B, lower panel). In contrast, no variation of TILs or PD-L1 expression were observed in the HLA-I–deficient tumors at relapse following BCG (Figure 7B, upper panel). However, we observed increased intratumoral CD163^+^ macrophages in those HLA-I–deficient tumors, consistent with our findings at the mRNA level (Figure 7B, pie charts in the upper panel), and which we did not observe in the HLA-I^+^ tumors (Figure 7B, pie charts in the lower panel). Of note, when we analyzed genes related to γ-δ T cells and NK cells (Supplemental Table 6), we found disparate variations but no significant change in gene expression levels across patients and could not find a clear association with their HLA-I tumor status (Supplemental Figure 7, F and G). Taken together, these data provide evidence that specific types of immune escape mechanisms are associated with the level of expression of HLA-I in tumors resistant or refractory to BCG immunotherapy.

Finally, we hypothesized that the level of HLA-I expression in tumors could impact the clinical outcome of patients with bladder cancer. To test this hypothesis, we identified which patients eventually developed distant metastasis or died from their bladder cancer in our cohort. With a 5-year follow-up, we found that 69% (*n =* 9 of 13) of the patients who had downregulated HLA-I levels in their tumor after BCG developed distant metastases (Figure 7, C and D). In contrast, only 14% (*n =* 2 of 14) of the patients developed metastases when HLA-I expression was maintained or upregulated in tumors at relapse after BCG during the same follow-up period. Furthermore, we observed a large difference in distant metastasis–free survival (DMFS), with a median DMFS of 27 months for patients with HLA-I^–^ tumors versus survival not reached for patients with HLA-I^+^ tumors (log rank *P =* 0.001). We also observed large differences in cancer-specific survival (CSS) and overall survival (OS) according to the tumor’s post-BCG HLA-I status. The median CSS and the median OS were both 37 months for HLA-I^–^ patients versus survival not reached for HLA-I^+^ patients (log rank *P =* 0.0006 and *P =* 0.003, respectively; Figure 7E). Taken altogether, this set of data illustrates that, at relapse, patients with downregulated HLA-I expression upon BCG therapy experience immune surveillance escape in their TME and have dismal outcomes.

## Discussion

Although conclusions from retrospective series of patient tumor samples can only be correlative, we show here that the HLA-I status of tumors that progress after intravesical BCG therapy defines 2 biologically distinct subgroups of patients with opposite outcomes. These observations suggest the existence of 2 different mechanisms of resistance to BCG therapy. In the first pattern, cancer cells have upregulated HLA-I protein expression upon BCG therapy and display increased numbers of CD3^+^CD8^+^ T cells in the TME, present a cytotoxic IFN-γ signature, and show T cell checkpoint upregulation. In the other pattern, tumors show downregulation of HLA-I protein expression upon BCG therapy, display no changes in T cells in their TME, and present an immunosuppressive myeloid profile. Our in vitro assays on fresh human bladder tumors and cancer cell lines demonstrated the causative effect of BCG on HLA-I downregulation by cancer cells, and the differential phenotype induced by BCG in terms of cytokine/chemokine and EMT profiles in cancer cells that have either upregulated or downregulated expression of their HLA-I molecules upon BCG exposure. Moreover, we show that live bacteria contained in clinical-grade BCG have the capacity to directly infect cancer cells. To the best of our knowledge, this capacity has only been demonstrated in vitro upon coincubation of live BCG strains expressing GFP (17). For the first time to our knowledge, we report this observation in our study using clinical-grade BCG, which contains a predominance of killed mycobacteria (18). Although live BCG is required to trigger the recruitment of T cells to the bladder microenvironment, the importance of the ratio of live versus dead mycobacteria has not been formally evaluated (11). Also, our results show that different human cancer cell lines have different sensitivities to BCG. One hypothesis, which would explain this observation, is that intracellular uptake of BCG by cancer cells could be dependent on their underlying level of kinase oncogenic stress. Indeed, using GFP-expressing BCG, Redelman-Sidi et al. demonstrated that BCG entry into cancer cells relies upon Rac1, Cdc42, and their effector kinase Pak1. They showed that cancer cell uptake of BCG is dependent on the oncogenic activation of signaling pathways such as those for phosphoinositide 3 kinase and RAS via micropinocytosis rather than phagocytosis (17).

Here, our data showed that intracellular *M. bovis*, the scientific strain name for BCG, induced a profound HLA-I downregulation in a subset of cancer cells, which led to the acquisition of EMT characteristics and limited inflammatory tumor responses in those cancer cells. Importantly, Carretero et al. previously identified more profound alterations of HLA-I expression in post-BCG recurrent tumors as compared with expression in pre-BCG tumors or tumors exposed to intravesical chemotherapy (19). Although this study included a small number of patients, to our knowledge, these data provide the first clinical evidence of immunotherapy-induced immune selection of HLA-I loss tumor variants in bladder cancer. Furthermore, our findings support the notion that 2 distinct patterns of tumor resistance with significant differences in outcomes occur upon BCG immunotherapy. Those biological and clinical fates seem to be associated with the regulation of HLA-I surface expression in cancer cells upon BCG exposure.

Several key conclusions can be drawn from these results. First, in ex vivo fresh human bladder tumors, we found that BCG had no effect on the activation phenotype of TILs, a finding consistent with observations made in an immune-competent orthotopic model of bladder cancer (20). However, our findings show that BCG induced a rapid (within 24 hours), profound, and sustained downregulation of HLA-I and β2m expression in ex vivo fresh human bladder tumors and in in vitro experimental data using several cancer cell lines. Interestingly, prior studies have shown that mycobacteria evade the host-adaptive immune response through multiple mechanisms. Specifically, live mycobacteria tuberculosis may escape immune control through inhibition of class I and class II MHC antigen processing in infected cells upon TLR signaling (21, 22). To our knowledge, the ability of live *M. bovis* BCG to infect cancer cells and orchestrate tumor immune suppression through HLA-I downregulation and a TLR-independent pathway has not previously been reported.

Importantly, we surmise that HLA-I downregulation in bladder cancers does not result from immunoediting but rather from an intracellular bacteria–induced EMT process that predicts a dismal prognosis. A lack of HLA-I expression by cancer cells is currently recognized as a hallmark of a cancer immunoediting process induced by TILs (23). Paradoxically, we found in our clinical cohort that half of the BCG-resistant tumors were associated with HLA-I upregulation and a Th1 type of tumor-infiltrative immune response. In contrast, we observed poor T cell infiltration in HLA-I–deficient tumors. Also, 2 distinct profiles of inflammatory cytokine/chemokine expression and secretion were identified in vitro when exposing the cancer cell lines or fresh bladder tumors to BCG. Those profiles were again associated with HLA-I expression levels in cancer cells. While HLA-I^+^ cancer cells released key effector cytokines and chemokines, such as CXCL10, CCL4, CCL5, and IFN-γ, HLA-I–deficient cancer cells had a limited release of proinflammatory cytokines. Building on these findings, we suggest that BCG can directly affect the immunogenicity of cancer cells, which subsequently orchestrate a distinct type of bladder cancer immune microenvironment. In situ immune profiling of BCG-resistant tumors strengthened this hypothesis. Specifically, HLA-I^+^ tumors displayed an in situ cytotoxic CD8^+^ T cell response, with the CXCL9/CXCL10/CXCL11/CXCR3 axis contributing to immune cell migration, activation, and differentiation. Additionally, the expression of multiple inhibitory receptors such as CTLA-4, TIGIT, LAG3, PD-1, and PD-L1 suggests that the tumor-acquired resistance was associated with T cell exhaustion in BCG-induced HLA-I^+^ tumors. By contrast, HLA-I–deficient tumors displayed an immunosuppressive microenvironment characterized by the upregulation of myeloid markers (CD163, CSF1R, CSF3R) and M2 macrophage–related cytokines (CCL18, IL-10) associated with CD8^+^ T cell exclusion from the TME. This observation led us to characterize a second mechanism of acquired resistance to BCG immunotherapy in HLA-I–deficient tumors. Our data support prior studies suggesting that immune suppression of the bladder microenvironment enriched with myeloid-derived suppressive cells (9) acquired upon BCG exposure may limit clinical-grade BCG efficacy and favor tumor recurrence. Hence, we identified 2 distinct patterns of immune escape following exposure to BCG and propose a biologically relevant and clinically feasible stratification strategy for the treatment of patients with BCG-unresponsive bladder tumors.

Of note, it might not be fortuitous that syngeneic and transplantable MB49 and UPPL1541 murine cancer cell lines were both found to be HLA-I^–^. Indeed, this feature may provide the immune escape mechanism required to have transplantable properties (invariably in males and females). Sun et al. showed also that MB49 cells were HLA-I^–^ and that exposure to BCG did not modify that status (24). Also de Queiroz et al. found that BCG, like TLR2, TLR4, and TLR9 agonists, could not trigger the secretion of proinflammatory cytokines from MB49 HLA-I^–^ murine bladder carcinoma (25). Mangsbo et al. showed that in vivo in mice, therapeutic strains of BCG were inactive against established MB49 bladder tumors (26). Altogether, the fact that the transplantable syngeneic murine cell lines are cells that have acquired a loss in HLA-I expression, an absence of cytokine secretion upon BCG exposure and a resistance to BCG therapy in vivo are consistent with our results obtained in the human context.

Last, our findings have established for the first time to our knowledge an unexpected association between a bacterial (BCG) intracellular infection of cancer cells and a cancer immune evasion mechanism with EMT characteristics. Our data showed a direct correlation between BCG exposure, HLA-I downregulation, EpCAM deficiency, and a shift toward a mesenchymal score in a subset of cancer cells. Of note, HLA-I downregulation was significantly more profound in BCG-infected cells and was associated with a type I IFN signaling pathway. A chronic type I IFN pathway has been recently described as one of the mechanisms of resistance to checkpoint blockade therapy (27). Also, the type I IFN gene signature has been established as a predictive factor for immune evasion leading to severe, uncontrolled active tuberculosis infection (28). Several mechanisms underlying the pathogenic role of type I IFN in tuberculosis have been described, including the induction of IL-10 cytokine expression and negative regulation of IL-12/IFN-γ and IL-1β/PGE2 host-protective responses, consistent with our in situ findings (29). In our clinical cohort, HLA-I–deficient tumors were associated with accelerated death due to distant metastasis, as opposed to HLA-I^+^ tumors, which were likely more immunogenic. This observation suggests that, upon BCG immunotherapy resistance, HLA-I–deficient cancer cells acquire an aggressive phenotype that increases the capacity for local invasion and distant metastasis. A prior study corroborates this hypothesis by showing that HLA-I downregulation is a shared feature of cancer cells with stem-like properties that are associated with tumor aggressiveness and a poor patient prognosis (30). Recently, in-depth analysis of tumor microbiota from multiple primary tumor sites revealed that both tumor cells and immune cells are infected by intracellular bacteria, with associations between specific bacteria, tumor subtypes, smoking status, and response to immunotherapy (31). Understanding how the tumor infection by bacteria amplifies or mitigates carcinogenesis and how it impacts the natural cancer immunosurveillance is currently an area of intensive interest (32).

Our results showing the impact of BCG on both HLA-I and autophagy must be put in the context of several related studies. First, *Mycobacteria tuberculosis* has been shown to interfere with the expression of HLA-I and the intracellular autophagy of infected cells (33). Second, the immune escape that occurs in pancreatic cancers has been recently related to HLA-I degradation via autophagy (34). Here, we provide evidence supporting the hypothesis that therapeutic BCG could induce HLA-I downregulation via autophagy flux inhibition. Therefore, drugs modulating autophagy could have a beneficial effect on HLA-I expression and favor the immune recognition of cancer cells.

New therapeutic strategies are currently in clinical development to treat BCG-unresponsive tumors, including the use of antagonistic Abs directed against the T cell immune checkpoints PD-1 and PD-L1, as well as oncolytic viruses and recombinant adenovirus IFN-α and stimulator of IFN gene (STING) agonists. Recently, the FDA has approved the anti–PD-1 mAb pembrolizumab for the treatment of BCG-unresponsive NMIBC. However, only a subset of patients seem to benefit from such PD-1–targeted therapy (35) in this setting, and BCG therapy remains the standard of care for first-line adjuvant therapy in high-risk NMIBC. Importantly, our findings have several specific translational implications. First, our study provides the rationale to stratify patients with BCG-resistant bladder tumors in 2 subgroups based on HLA-I tumor phenotype. Indeed, pathologists can easily assess HLA-I expression in bladder tumors by IHC staining, as this assay is already done in routine practice. Second, the segregation of BCG-resistant tumors according to HLA-I phenotype would offer an opportunity for adapted clinical care of BCG-unresponsive bladder cancers. Dedicated clinical trials for treatment intensification should now be designed for HLA-I–deficient tumors to improve the survival of this new subgroup of patients. Treatment intensification may include therapeutics addressing the typical immune suppression described in the HLA-I–deficient bladder cancers. Indeed, those HLA-I–deficient tumors should not be recognized by CD8^+^ T cells and therefore would not benefit from checkpoint blockade immunotherapies. Such HLA-I^–^ cancers could therefore benefit from therapies such as autophagy modifiers or T cell–engaging Abs (HLA-I requirement bypass). Conversely, patients with HLA-proficient tumors show upregulation of several immune checkpoint–inhibitory receptors (CTLA-4, TIGIT, LAG3, PD-1) and the ligand PD-L1, which suggests that immune checkpoint blockers such as anti–CTLA-4, anti-TIGIT, or anti-LAG3 mAbs may be active in BCG-unresponsive HLA-I^+^ tumors. Also, treatment strategies combining first-line intravesical BCG with systemic anti–PD-L1 mAbs are currently under investigation in the adjuvant setting (NCT03528694 and NCT03799835). Our results show that only patients without downregulation of HLA-I in their tumor upon BCG exposure would benefit from the addition of such anti–PD-1 or anti–PD-L1 mAbs.

Overall, our results offer a simple opportunity for patient stratification upon BCG immunotherapy failures based on tumor HLA-I status and provide the rationale for further investigation of distinct therapeutic strategies for those patients with HLA-I^+^ or HLA-I^–^ tumors.

## Methods

Additional information can be found in Supplemental Methods.

### BCG reconstitution.

Bacteria used for in vitro experiments was obtained by resuspension in PBS of the lyophilized content of clinically available ImmuCyst vials.

### Ex vivo bladder tumor stimulation assay.

Freshly dissociated cells from human bladder tumors were seeded in a 96-well plate and incubated for 72 hours with or without BCG. Cells were harvested, followed by membrane and intracellular labeled according to the manufacturer’s protocol, before running them through the flow cytometer.

### Cell lines.

All human bladder cancer cell lines including RT4, SW780, HT1376, 5637, TCCSUP, and UM-UC3 were purchased from the American Type Culture Collection (ATCC, no. TCP-1020). The human A549 cell line was obtained from Nicolas Dorvault (INSERM U981, Gustave Roussy Comprehensive Cancer Center, Université Paris-Saclay, Villejuif, France). The human HeLa, HCT116, and Mel888 cell lines were obtained from Laurence Zitvogel (INSERM U1015, Gustave Roussy Comprehensive Cancer Center, Université Paris-Saclay, Villejuif, France). The murine bladder cancer cell line MB49 was obtained from MilliporeSigma (catalog SCC148). The UPPL bladder cancer cell line was obtained from William Y. Kim (Lineberger Comprehensive Cancer Center, University of North Carolina at Chapel Hill, Chapel Hill, North Carolina, USA).

### Cytokine and chemokine measurement.

Titration of cytokines and chemokines was performed using the Bio-Plex Pro Assays Chemokine Quick Guide protocol (Bio-Rad 27-Plex Human Chemokine Panel, kit no. M500KCAF0Y). This kit detects IL-1β, IL-1ra, IL-4, IL-5, IL-6, IL-7, IL-8, IL-10, IL-12 (p70), IL-13, IL-15, IL-17, TNF-α, IFN-γ, CXCL10, G-CSF, GM-CSF, eotaxin, CCL2, CCL3, CCL4, CCL5, VEGF, FGF basic, and PDFG-bb. Acquisitions were performed on a Bio-Plex 200 system (Bio-Rad), and analyses were done using Bio-Plex Manager 6.1 Software (Bio-Rad), respectively.

### Flow cytometric analyses.

For membrane labeling, TILs and cancer cells were stained with fluorochrome-coupled mAbs incubated for 20 minutes at 4°C, protected from light, and then washed with 1× PBS. Intracellular staining was performed after permeabilization with the FoxP3 Transcription Factor Staining Buffer Set (Thermo Fisher Scientific, catalog 00-5523-00) and intracellularly labeled with anti-Foxp3 mAb (eBiosciences, clone PCH101) and anti-Ki67 (BD Biosciences, catalog 556027), following the manufacturer’s protocol. Cell samples were acquired on a BD LSRFortessa X-20 flow cytometer (BD Biosciences) with single-stained Ab-capturing beads used for compensation (CompBeads, BD Biosciences, catalog 552843). For cell sorting, Zombie Aqua^–^ cells and anti-HLA-I^+^ and HLA-I^–^ cells after BCG coincubation for 24 hours were sorted on a BD FACSAria III Fusion (BD Biosciences). Data were analyzed with Kaluza Analysis software, version 2.1 (Beckman Coulter). MB49 and UPPL1541 cell lines were cultured in vitro into media with or without recombinant mouse IFN-γ (BioLegend, catalog 575306) for 24 hours and subsequently stained for HLA-I with FITC anti-mouse H-2Kb Ab (BioLegend, catalog 1165005) and run by flow cytometry.

### In vitro BCG restimulation assay.

Cancer cells (250,000 cells/well) were added to flat-bottomed, 6-well plates. After incubation for 24 hours at 37°C and washing with warm PBS, cancer cells were incubated with culture medium only as a negative control (2000 μL/well RPMI plus 10% heat-inactivated FBS) or BCG at a MOI of 10:1, for 24 hours. After 24 hours, the cells were washed twice with 500 μL warm 1× PBS and incubated for 6 days in culture medium with 10% serum, and the medium was changed once on day 3. Cells were harvested on day 5 using the trypsin protocol, washed twice with 1× PBS, and seeded (250,000 cells/well) in flat-bottomed, 6-well plates for 24 hours. Cells were restimulated with BCG at a MOI of 10:1. After 24 hours, supernatants were collected and stored at –20°C until cytokine measurement.

### Immunofluorescence.

Bladder cancer cells (5637 cell line) were grown on coverslips for 1 day before a 24-hour infection with BCG at a MOI of 10:1. Cells were washed once in PBS and fixed for 20 minutes in 4% PFA. After 3 washes in PBS, cells were blocked for 30 minutes in PBS 3% BSA at room temperature (RT). Then, nuclei were stained for 5 minutes in a 100 ng/μL DAPI solution. HLA-ABC primary Ab (Thermo Fisher Scientific, MA5-11723) was diluted up to 10 μg/μL in PBS 3% BSA and incubated overnight. After 3 washes in PBS, cells were incubated in a 1:1000 dilution of goat anti–mouse secondary Ab coupled with Alexa Fluor Plus 555 (Thermo Fisher Scientific, A32727). Cells were washed 3 times and mounted in ProLong Gold (Thermo Fisher Scientific, P36930). Confocal images were acquired on a Leica SP8, and images were analyzed with Fiji 64 bits.

### Chromogenic IHC.

Consecutive 3 μm thick sections of formalin-fixed, paraffin-embedded (FFPE) tumor tissues were cut on a microtome and floated on a 40°C water bath containing distilled water. Sections were transferred onto positively charged glass slides (Superfrost Plus). Slides were dried overnight and stored at 4°C until use. Consecutive FFPE slides were stained using validated and standardized protocols on Ventana Discovery Ultra or Benchmark Ultra-Automated platforms (Roche Diagnostics, Ventana Medical Systems).

### IHC analysis for HLA-I and PD-L1 expression.

For HLA-1 (clone EMR 8-5 anti–HLA class I heavy chains A, B, and C), the intensity of tumor cell membrane staining was scored using the following semiquantitative scale: 0 (negative), 1 (weak), 2 (moderate), and 3 (strong). Then, the percentage of positively stained tumor cells was multiplied by the staining intensity of the tumor cell to obtain a final semiquantitative H score (score of 0–300). For PD-L1 staining (E1L3N clone, Cell Signaling Technology), primary Ab was incubated at a 1 μg/mL dilution for 1 hour at RT. Detection was performed using a HQ amplification kit and 3,3′-diaminobenzidine as a chromogen. For each tumor sample, PD-L1 expression was scored by a pathologist trained in tumor cell analysis (percentage of cells with membrane staining) and immune cells (percentage of tumor areas covered by PD-L1–expressing tumor cells).

### Image analysis.

Image acquisition was performed on a Virtual Slide Microscope VS120-SL (Olympus) with a ×20 air objective (0.75 NA, 345 nm/pixel). CD3^+^ and CD8^+^ lymphocytes were detected using an algorithm created in Definiens Developer software. Macrophages results are expressed as the area of CD163^+^ and CD68^+^CD163^–^ stained cells divided by the region of interest (ROI) area.

### NanoString gene expression profiling.

Isolated RNA from a human tumor specimen and cell lines were hybridized with the NanoString nCounter PanCancer Immune Profiling Human Panel CodeSet and the NanoString nCounter IO360 Panel Human Panel CodeSet, respectively. RNA was quantified using the nCounter Digital Analyzer. Data were processed with nSolver Analysis Software (NanoString) using the Advanced Analysis module.

### Transcriptomics (3′tag-Seq).

RNA-Seq was performed on FFPE tumor RNA (100 ng) from 14 patients at baseline using 3′ Tag-Seq. We used Lexogen’s QuantSeq 3′ mRNA-Seq Library Prep Kit (Illumina). Transcriptomic data have been deposited in the NCBI’s Gene Expression Omnibus (GEO) database (GEO GSE199618).

### Bioinformatics analysis.

For quantification of gene expression, STAR was used to obtain the number of reads associated with each gene in the Gencode version 31 annotation (restricted to protein-coding genes, antisense, and long, intergenic noncoding RNAs [lincRNAs]). Raw counts for each sample were imported into R statistical software. The extracted count matrix was normalized to the library size to compute counts per million reads mapped (CPM) expression levels. Differential gene expression analyses in paired tissue samples before and after BCG have identified genes associated with outcomes. The Bioconductor edgeR package was used to import raw counts into R statistical software. Differential expression analysis was performed using the Bioconductor limma package and the voom transformation.

### ImageStream analysis.

Images were captured on an Amnis ImageStream Mark II Imaging Flow Cytometer with ×40 magnification (EMD Millipore). Data were acquired and analyzed using Amnis INSPIRE software and Amnis IDEAS software, respectively.

### Quantification of autophagy in U2OS cells for in vitro studies.

Human osteosarcoma U2OS cells stably expressing GFP-LC3 or RFP-GFP-LC3 (PMID 21151176) were seeded at 1500 cells/well in μClear imaging plates (Greiner BioOne, Kremsmünster, Austria) and left to adapt for 24 hours before adding BCG at a MOI of 10:1, 30:1, 100:1, and 300:1. Torin (Tocris) at 0.3 μM was used as a positive control for autophagy induction. After 6 hours of incubation, cells were fixed with 3.7% paraformaldehyde (PFA) (w/v in PBS) containing 1 μg/mL Hoechst 33342 for 30 minutes. The fixative was then removed, the cells were overlaid with PBS, and the plates were sealed with adhesive aluminum tape. Images were acquired by means of an IXMc confocal automated bioimager (Molecular Devices) equipped with a ×20 PlanApo objective (Nikon). Images were processed with R.

### Statistics.

Statistical tests were calculated in GraphPad Prism, version 8.0 (GraphPad Software). A paired, 2-tailed Student’s *t* test was used to compare 2 groups unless indicated otherwise, and 1-way ANOVA was used to compare more than 2 groups with 1 independent variable, followed by Tukey’s correction for multiple comparisons. For Kaplan-Meier survival experiments, we performed a log-rank (Mantel-Cox) test. A *P* value of less than 0.05 was considered statistically significant.

### Study approval.

Paired tumor samples were collected from patients with high-grade NMIBC who received standard-of-care transurethral resection of the bladder tumor (TURBT) and intravesical BCG therapy at the University Paris-Saclay Hôpital Foch between January 2000 and May 2015, as previously reported (36). Study of this retrospective cohort and the associated informed consent procedures were approved by the institutional ethics committee of Hôpital Foch (IRB approval no. 00012437). In situ multidimensional profiling data (chromogenic IHC, NanoString gene expression profiling, and RNA-Seq) were generated using paired samples from pre- and post-BCG treatment tumors. Multidimensional profiling data were analyzed to identify immune correlates of BCG resistance mechanisms (Supplemental Figure 6 and Supplemental Table 5). Fresh tumor samples were prospectively collected from specimens obtained from radical cystectomy procedures (*n =* 12) performed with curative intent at the UVSQ – University Paris-Saclay Hôpital Foch between April 2018 and September 2018. This prospective cohort was approved by the institutional ethics committee (study “mAb in vitro test,” no. ID-RCB: 2016-A00732-49).

## Author contributions

MR, TL, JCS, LZ, and AM conceived and designed the study. Acquisition of data: MR, JA, CR, SM, DB, NS, AB, MD, AKS, RC, SV, AGG, DL, ML, and NC acquired data. All authors contributed to the analysis and interpretation of data and to critical revision of the manuscript. MR wrote the manuscript. MR and TZT performed statistical analyses. DB provided administrative and technical support, and CR provided material support.

## Supplementary Material

Supplemental data

## Version 1 05/03/2022

In-Press Preview

## Version 2 06/15/2022

Electronic publication

## References

[1] Hedegaard J (2016). Comprehensive Transcriptional Analysis of Early-Stage Urothelial Carcinoma. Cancer Cell.

[2] Tan TZ (2019). Molecular subtypes of urothelial bladder vancer: results from a meta-cohort analysis of 2411 tumors. Eur Urol.

[3] Lamm DL (1991). A randomized trial of intravesical doxorubicin and immunotherapy with bacille calmette-guérin for transitional-cell carcinoma of the bladder. N Engl J Med.

[4] Brosch R (2007). Genome plasticity of BCG and impact on vaccine efficacy. Proc Natl Acad Sci U S A.

[5] Chamie K (2013). Recurrence of high-risk bladder cancer: a population-based analysis. Cancer.

[6] Van Den Bosch S, Witjes JA (2011). Long-term cancer-specific survival in patients with high-risk, non-muscle-invasive bladder cancer and tumour progression: a systematic review. Eur Urol.

[7] Pietzak EJ (2019). Genomic differences between “primary” and “secondary” muscle-invasive bladder cancer as a basis for disparate outcomes to cisplatin-based neoadjuvant chemotherapy. Eur Urol.

[8] Kamat AM (2016). Cytokine panel for response to intravesical therapy (CyPRIT): nomogram of changes in urinary cytokine levels predicts patient response to bacillus Calmette-Guérin. Eur Urol.

[9] Chevalier MF (2017). ILC2-modulated T cell-to-MDSC balance is associated with bladder cancer recurrence. J Clin Invest.

[10] Pichler R (2017). Intratumoral Th2 predisposition combines with an increased Th1 functional phenotype in clinical response to intravesical BCG in bladder cancer. Cancer Immunol Immunother.

[11] Biot C (2012). Preexisting BCG-specific T cells improve intravesical immunotherapy for bladder cancer. Sci Transl Med.

[12] Antonelli A (2020). Bacterial immunotherapy for cancer induces CD4-dependent tumor-specific immunity through tumor-intrinsic interferon-γ signaling. Proc Natl Acad Sci.

[13] Kates M (2020). Adaptive immune resistance to intravesical BCG in non–muscle invasive bladder cancer: implications for prospective BCG-unresponsive trials. Clin Cancer Res.

[14] Kobayashi T (2015). Modelling bladder cancer in mice: opportunities and challenges. Nat Rev Cancer.

[15] Tan TZ (2014). Epithelial-mesenchymal transition spectrum quantification and its efficacy in deciphering survival and drug responses of cancer patients. EMBO Mol Med.

[16] Galluzzi L (2012). Trial watch:experimental toll-like receptor agonists for cancer therapy. Oncoimunnology.

[17] Redelman-Sidi G (2013). Oncogenic activation of Pak1-dependent pathway of macropinocytosis determines BCG entry into bladder cancer cells. Cancer Res.

[18] Behr MA (2002). BCG--different strains, different vaccines?. Lancet Infect Dis.

[19] Carretero R (2011). Bacillus Calmette-Guerin immunotherapy of bladder cancer induces selection of human leukocyte antigen class I-deficient tumor cells. Int J Cancer.

[20] Kates M (2017). Intravesical BCG Induces CD4^+^ T-cell expansion in an immune competent model of bladder cancer. Cancer Immunol Res.

[21] Tobian AAR (2003). Alternate class I MHC antigen processing is inhibited by toll-like receptor signaling pathogen-associated molecular patterns: *Mycobacterium tuberculosis* 19-kDa lipoprotein, CpG DNA, and lipopolysaccharide. J Immunol.

[22] Harding CV, Boom WH (2010). Regulation of antigen presentation by *Mycobacterium tuberculosis*: a role for Toll-like receptors. Nat Rev Microbiol.

[23] Schreiber RD (2011). Cancer immunoediting: integrating immunity’s roles in cancer suppression and promotion. Science.

[24] Sun E (2015). Recombinant hIFN-α2b-BCG inhibits tumor growth in a mouse model of bladder cancer. Oncol Rep.

[25] de Queiroz NMGP (2021). MyD88-dependent BCG immunotherapy reduces tumor and regulates tumor microenvironment in bladder cancer murine model. Sci Reports.

[26] Mangsbo SM (2008). CpG therapy is superior to BCG in an orthotopic bladder cancer model and generates CD4^+^ T-cell immunity. J Immunother.

[27] Benci JL (2016). Tumor interferon signaling regulates a multigenic resistance program to immune checkpoint blockade. Cell.

[28] Singhania A (2018). The value of transcriptomics in advancing knowledge of the immune response and diagnosis in tuberculosis. Nat Immunol.

[29] Lúcia Moreira-Teixeira (2018). Type I interferons in tuberculosis: Foe and occasionally friend. J Exp Med.

[30] Domingo-Domenech J (2012). Suppression of acquired docetaxel resistance in prostate cancer through depletion of notch- and hedgehog-dependent tumor-initiating cells. Cancer Cell.

[31] Nejman D (2020). The human tumor microbiome is composed of tumor type-specific intra-cellular bacteria. Science.

[32] Zitvogel L (2016). Microbiome and anticancer immunosurveillance. Cell.

[33] Strong EJ, Lee S (2021). Targeting autophagy as a strategy for developing new vaccines and host-directed therapeutics against mycobacteria. Front Microbiol.

[34] Yamamoto K (2020). Autophagy promotes immune evasion of pancreatic cancer by degrading MHC-I. Nature.

[35] Gill J, Prasad V (2021). Pembrolizumab for non-muscle-invasive bladder cancer-a costly therapy in search of evidence. JAMA Oncol.

[36] Rouanne M (2019). Stromal lymphocyte infiltration is associated with tumour invasion depth but is not prognostic in high-grade T1 bladder cancer. Eur J Cancer.

