# Supplementary material for: BCG therapy downregulates HLA-I on malignant cells to subvert antitumor immune responses in bladder cancer
Source: J Clin Invest. 2022 Jun 15;132(12):e145666. doi: 10.1172/JCI145666 (PMC9197524; doi:10.1172/JCI145666)
Supplement: Supplemental data [file jci-132-145666-s111.pdf]

Supplemental Materials for

BCG therapy downregulates HLA-I on malignant cells to subvert antitumor immune responses in bladder cancer

Authors:

Mathieu Rouanne*[1,2,3], Julien Adam[4,5†], Camélia Radulescu[6], Diane Letourneur[1,7], Séverine Mouraud[1], Delphine Bredel[1], Anne-Gaëlle Goubet[1], Marion Leduc[8,18], Noah Chen[2], Tuan Zea Tan[9], Nicolas Signolle[10], Amélie Bigorgne[11,12], Michael Dussiot[11], Lambros Tselikas[1], Sandrine Susini[1], François-Xavier Danlos[1], Anna K. Schneider[10‡], Roman Chabanon[13,14], Sophie Vacher[15], Ivan Bièche[15], Thierry Lebret[3], Yves Allory[6,15,16], Jean-Charles Soria[12,18], Nicholas Arpaia[2,19], Guido Kroemer[8,18,20,21], Oliver Kepp[8,18], Jean Paul Thiery[10,22], Laurence Zitvogel[1,23], Aurélien Marabelle*[1,12,23]

Affiliations :

[1]*Institut National de la Santé et de la Recherche Médicale (INSERM) U1015, Equipe Labellisée Ligue Nationale contre le Cancer, Gustave Roussy, Université Paris-Saclay, 94805 Villejuif, France*

[2]*Department of Microbiology and Immunology, Vagelos College of Physicians and Surgeons, Columbia University, New York, NY 10032, USA*

[3]*Département d'Urologie, Hôpital Foch, UVSQ-Université Paris-Saclay, 92150 Suresnes, France*

[4]Département de Biologie et Pathologie Médicales, Gustave Roussy, Université Paris-Saclay, 94805 Villejuif, France

[5]INSERM U1186, Gustave Roussy, 94805 Villejuif, France

[6]Département de Pathologie, Hôpital Foch, UVSQ-Université Paris-Saclay, 92150 Suresnes, France

[7]Master de Biologie, École Normale Supérieure de Lyon, Université Claude Bernard Lyon I, Université de Lyon, 69342, Lyon Cedex 07, France

[8]Metabolomics and Cell Biology Platforms, Gustave Roussy Cancer Center, Université Paris Saclay, 94805 Villejuif, France

[9] Genomics and Data Analytics Core (GeDaC), Cancer Science Institute of Singapore, National University of Singapore, 14 Medical Drive, #12-01, Singapore 11759

[10]INSERM U981, Gustave Roussy, 94805 Villejuif, France

[11]INSERM U1163, Institut Imagine, Université de Paris, 75015 Paris, France

[12]Département d'Innovation Thérapeutique et d'Essais Précoces (DITEP), Gustave Roussy, Université Paris-Saclay, 94805 Villejuif, France

[13]ATIP-Avenir Group, INSERM U981, Gustave Roussy, Université Paris-Saclay, 94805 Villejuif, France

[14]The CRUK Gene Function Laboratory and Breast Cancer Now Toby Robins Breast Cancer Research Centre, The Institute of Cancer Research, London, United Kingdom

[15]Service de Génétique, Institut Curie, PSL Research University, 75005 Paris, France.

[16]Départment de Pathologie, Institut Curie, 92210 Saint-Cloud, France

[17]Centre National de la Recherche Scientifique (CNRS) UMR144, Equipe Labellisée Ligue Nationale Contre le Cancer, 75005 Paris, France

[18]*Gustave Roussy, Université Paris-Saclay, 94805 Villejuif, France*

[19]*Herbert Irving Comprehensive Cancer Center, Columbia University, New York, NY 10032, USA*

[20]*Centre de Recherche des Cordeliers, Equipe labellisée par la Ligue contre le cancer, Université de Paris, Sorbonne Université, INSERM U1138, Institut Universitaire de France, Paris, France*

[21]*Pôle de Biologie, Hôpital Européen Georges Pompidou, AP-HP, Paris, France*

[22]*Guangzhou Laboratory, Guangzhou 510530, China*

[23]*Centre d'Investigation Clinique de Biothérapies du Cancer (CICBT), 94805 Villejuif, France*

†Present address: Urology Research Unit, Departement of Urology, Centre Hospitalier Universitaire Vaudois (CHUV), 1011 Lausanne, Switzerland.

‡Present address: Department of Pathology, Saint Joseph Hospital, 75014 Paris, France

Correspondance to:

Dr Mathieu Rouanne MD, PhD
Email: rouanne.mathieu@gmail.com; mr4104@cumc.columbia.edu

Prof. Aurélien Marabelle MD, PhD

Email: aurelien.marabelle@gustaveroussy.fr

Supplemental Methods

Patient cohort and clinical end points

A cohort of 27 patients with non-metastatic, primary high-grade NMIBC was included in this study. Patients who acquired BCG resistance defined as tumor progression to muscle-invasive tumor (MIBC) post-BCG were included. Clinical follow-up included cystoscopy and urine cytology every 3 months for the first 2 years, then every 6 months. Tumor samples were obtained by TURBT or radical cystectomy and provided formalin-fixed and paraffin-embedded (FFPE). Only initial high-grade tumors with a visible, clearly identifiable, and disease-free muscularis propria were included in this study. Tumor samples obtained pre-BCG at baseline and post-BCG at time of disease progression was required for enrollment. Importantly, the FFPE blocks were selected from the first TURBT to avoid post-surgical inflammatory lesions in bladder tissue wound repair (Supplemental Figure 6). Tumor samples were initially reviewed by two pathologists (C.R. and J.A.). Clinicopathological and demographic data were collected from patient records at Hôpital Foch and are shown in Supplemental Table 5. Overall survival (OS) was defined as the time between the TURBT and the date of death (any cause). Cancer-specific survival (CSS) was defined as the time between the first TURBT and the date of death related to bladder cancer. For subjects without documentation of death, OS and CSS were censored on the last date the patient was known to be alive. Distant metastasis free survival (DMFS) was defined as the time between the first TURBT and the date of documented distant metastasis. For patients without documentation of distant metastasis, DMFS was censored on the last date the patient was known to be without metastasis.

Fresh tumor samples

All the tumors were collected within 1hr from surgery and stored in complete medium at 4°C from 12hrs to 20hrs prior to dissociation. Clinicopathological and demographic data were collected from patient records at Hôpital Foch and are shown in Supplemental Table 1. Primary bladder tumors were freshly mechanically and enzymatically dissociated using the Miltenyi Gentle MACS OctoDissociator (Miltenyi Biotec) equipment for 1hr at 37°C under mechanical rotation (2 incubation steps of 30 minutes).

The medium used for tumor dissociation consisted of RPMI 1640 (GIBCO Life Technologies, ref: 31870-025), Collagenase IV (50IU/mL) (Sigma-Aldrich, ref: C2139), Hyaluronidase (280IU/mL) (Sigma-Aldrich, ref: H6254), and DNAse I (30IU/mL) (Sigma-Aldrich, ref: 260913). Cell samples were diluted in PBS, passed through a cell strainer, and centrifuged for 5 minutes at 1500 rpm. Cells were finally resuspended in PBS, stained for baseline phenotyping of $CD45^+$ immune cells and counted with Precision Count BeadsTM (Biolegend, ref: 424902) by flow cytometry following manufacturer's protocol.

BCG reconstitution

ImmuCyst BCG is made from a live-attenuated strain of *Mycobacterium bovis*. The *bacilli* are lyophilized (freeze-dried) and are viable upon reconstitution. It contains 81 mg (dry weight) of BCG and 150 mg monosodium glutamate. Each vial of ImmuCyst is reconstituted with 3 mL of sterile, preservative-free saline solution. The reconstituted dose contains approximately $10.5 \pm 8.7 \times 10^8$ colony-forming units (CFU). For clinical use, the reconstituted material from the vial is further diluted in an additional 50 mL of sterile, preservative-free saline solution to a final volume of 53 mL for instillation into the bladder. Therefore, the final concentration in the bladder is approximately 2×10^7 CFU/mL.

Ex vivo bladder tumor stimulation assay

The experimental protocol was adapted from Jacquelot et al (37). We performed an ex vivo tumor stimulation assay providing from 12 fresh human bladder tumors. Freshly dissociated cells were seeded in 96-wells plate and incubated in complete medium (RPMI 1640 (GIBCO Life Technologies, ref: 31870-025) supplemented with 10% human AB serum (Institute Jacques Boy, ref: 201021334), 1% Penicillin/Streptomycin (GIBCO Invitrogen, ref:15140-122), 1% L-glutamine (GIBCO Life Technologies, ref: 25030-024) and 1% of sodium pyruvate (GIBCO Life Technologies, ref: 11360-039)), or stimulated with IFNg (1.10^3 UI/mL) or with reconstituted ImmuCyst (BCG sub-strain Connaught 2.10^7 CFU/mL).

Cell lines

Murine bladder cancer cell line MB49 was originally generated by 7,12-dimethylbenz[a]anthracene (DMBA) in vitro exposure to bladder epithelium from male C57BL/Icrf mice (38). The UPPL bladder cancer cell line was obtained from Prof William Y. Kim of the University of North Carolina. UPPL1541 was originally generated from spontaneously arising bladder tumors in Upk3a-Cre^{ERT2}; Trp53$^{L/L}$; Pten$^{L/L}$; Rosa26$^{LSL\text{-}Luc}$ mice (39). All the cell lines have been tested and found negative for Mycoplasma contamination.

BCG co-culture

Urothelial cancer cells were plated a day prior to infection in antibiotic-free media to reach 80-90% confluence on the day of infection. Lyophilized ImmuCyst Bacillus Calmette-Guérin (BCG) (Connaught sub-strain, 81 mg at 10.5 ± 8.7 10^8 CFU/mL) was reconstituted within PBS 1X as recommended for clinical use. BCG was co-incubated in antibiotic-free media to achieve a multiplicity of infection (MOI) of 10:1 or 5:1 as reported. Plates were

incubated at 37°C for the indicated time and then washed with 1XPBS, detached using trypsin, resuspended in complete medium. After passage on 70µm filter and centrifugation, cells were resuspended in 1XPBS for analysis by flow-cytometry.

***In vitro* BCG re-stimulation assay**

This protocol originally described to study trained immunity in human monocytes has been adapted from Bekkering et al (40).

Chromogenic Immunohistochemistry (IHC)

The main steps for chromogenic IHC are described below. Sections were deparaffinized in xylene. Antigen retrieval was performed using ultra cell conditioning 1 (CC1) buffer for 36 minutes at 95°C. Sections were incubated with a primary antibody (see table below) during 1hr at room temperature. Amplification was achieved using an UltraView universal DAB detection kit. Revelation using 3,3'diaminobenzidine as chromogen was applied to sections. Nuclear counterstaining was performed with Hematoxylin II and bluing reagent. Coverslip was applied with a permanent mounting medium.

Image analysis

Image analysis was done on manually selected (exclusion of areas of necrosis, preparation artifacts) regions of interest (ROI). As these regions were large, they were divided into blocks of pixels processed individually and stitched at the end. The method combined watershed segmentation on DAB staining and color and morphological characteristics to retrieve automatically $CD3^+$ or $CD8^+$ cells. The program exports the number of $CD3^+$ or $CD8^+$ cells and the tissue areas in $µm^2$ for each analyzed ROI. DAB-stained nucleus is automatically detected using their IHC spectral properties in manually

selected regions of interest. The routine scores (low, medium, and high) each nucleus on its intensity. In our cases, low classification corresponded to false positive staining and was discarded.

NanoString gene expression profiling

Formalin-fixed paraffin-embedded tumor specimens with sufficient bladder tumor area were selected for RNA extraction (n=12). Only paired bladder samples pre- and post-BCG therapy were used for this study. Macrodissection of selected tumor areas followed by RNA extraction using High Pure FFPET RNA Isolation Kit – Roche Life Science (ref:06483852001) were performed. The samples were stored at –80°C. Isolated RNA was hybridized with the NanoString nCounter PanCancer Immune Profiling Human Panel CodeSet. For cancer cell analysis, cell sorting was performed 24hrs after BCG co-culture. Next, RNA was extracted using the Direct-zol RNA MiniPrep Kit (Zymo Research). The samples were stored at –80°C. Isolated RNA was hybridized with the NanoString nCounter IO360 Panel Human Panel CodeSet and quantified using the nCounter Digital Analyzer. Data were processed with nSolver Analysis Software (NanoString) using the Advanced Analysis module.

Transcriptomics (3'Tag-Seq)

In contrast to traditional RNA-seq, which generates sequencing libraries for the whole transcript, 3′ Tag-Seq only generates a single initial library molecule per transcript, complementary to 3′ end sequences. In consequence, for human samples the restriction to a small part of the transcripts reduces the number of sequencing reads required, has exceptionally low background noise, as well as insensitivity to RNA sample quality variations, in particular FFPE tumor samples.

Bioinformatics analysis

To improve the statistical power of the analysis, only genes expressed in at least one sample (CPM >= 0.1) were considered. A qval threshold of <= 0.05 and a minimum fold change of 1.2 were used to define differentially expressed genes. Pathway enrichment analysis – GSEA Gene list from the differential analysis was ordered by decreasing log2 fold change. Gene set enrichment analysis was performed by clusterProfiler:GSEA function using the fgsea algorithm.

ImageStream analysis

For in vitro infection, BCG was labelled with calcein after co-incubation in complete medium for 30 min at 37°C 5%CO_2 protected from light. Urothelial cancer cells were co-incubated with calcein-labeled BCG (MOI 10:1) for 24hrs or incubated with IFNg (1.10^3 U/mL) or RMPI 10% heat-inactivated FBS. Cells were harvested using trypsin, washed twice with 1xPBS, and stained with surface antibodies. Hoeschst was added 15 min before image acquisition.

Quantification of autophagy in U2OS cells (image analysis)

Images were processed and segmented with R using the EBImage package (available on the Bioconductor repository https://www.bioconductor.org), the MorphR and the MetaxpR package (both available at https://github.com/kroemerlab). First the nuclear region was defined by using a polygon mask based on the fluorescent signal of Hoechst 33342, which allowed for the further segmentation of cells and for evaluating morphological parameters such as the size of the nucleus and the Hoechst signal intensity. Extending from this nuclear region of interest (ROI), the cytoplasmic region was

identified based on the diffuse GFP signal present in the biosensor cells expressing GFP-LC3. Cytoplasmic regions of higher GFP fluorescens were detected to quantify GFP-LC3 puncta formation. For the assessment of autophagic flux the LC3 puncta were additionally detected based on the RFP signal.

Quantification of autophagy in U2OS cells (data analysis)

Data extracted from image analysis were further analyzed with the R software. First pyknotic nuclei, dead cells and debris were excluded from the data set, based on intensity and size parameters. GFP-LC3 aggregation was evaluated by the number/surface area of GFP dots per cell; data were normalized using negative control (untreated condition) to obtain a fold change. Autophagic fluxes were assessed using the surface of autophagosomes compared to the total surface of aggregates (autophagosomes and autophagolysosomes). A linear regression between the surface of dots present in both channels (autophagosomes only) and the total surface of dots (autophagosomes and autophagolysosomes) was calculated on control data (negative and positive control data), and a flux inhibition score was computed by calculating the distance of each data point to the regression line. Results were then evaluated based on inhibition score and the total surface of aggregates.

List of antibodies used in this study

Antigen	Conjugate	Clone	Vendor	Catalog number
HLA-I	AF700	W6/32	BioLegend	311438
CD45	BUV805	HI30	BD Biosciences	564914
EpCAM	PECy7	1B7	eBiosciences	25-9326-42
Zombie Aqua	BV510	N/A	Biolegend	423102
Annexin-V	APC	N/A	Biolegend	640920

Ki-67	FITC	N/A	BD Biosciences	556026
CD3	BUV395	UCHT1	BD Biosciences	563546
CD4	BUV496	SK3	BD Biosciences	564651
FoxP3	APC	PCH101	eBiosciences	17-4776-42
CD8	APC-H7	SK1	BD Biosciences	560179
CD20	PE	2H7	Biolegend	302306
CD11b	PB	M1/70	Biolegend	101224
CD56	BV650	HCD56	Biolegend	318344
HLA-DR	PerCpCy5.5	G46-6	BD Biosciences	552764
CD69	FITC	FN50	BD Biosciences	555530
PD1	PECy7	PD1.3	Beckman	PN A78885
Mouse H-2Kb	FITC	AF6-88.5	BioLegend	116505

Supplemental References

37. Jacquelot N et al. Predictors of responses to immune checkpoint blockade in advanced melanoma. *Nat. Commun.* 2017;8(1):592.

38. Franks ISL. Effects of donor age on neoplastic transformation of adult mouse bladder epithelium in vitro. *JNCI J. Natl. Cancer Inst.* 1979;62(4):1017–1023.

39. Saito R et al. Molecular Subtype-Specific Immunocompetent Models of High-Grade Urothelial Carcinoma Reveal Differential Neoantigen Expression and Response to Immunotherapy. *Cancer Res.* 2018;78(14):3954–3968.

40. Bekkering S et al. In Vitro experimental model of trained innate immunity in human primary monocytes. *Clin. Vaccine Immunol.* 2016;23(12):926–933.

Supplemental Figures

Supplemental Figure 1. Ex vivo BCG stimulation of fresh human bladder tumors and cancer cell lines induces HLA-I downregulation on cancer cells.

Supplemental Figure 2. BCG induces EMT characteristics in HLA-I$^-$ cancer cells

Supplemental Figure 3. BCG mostly induces inflammatory responses in HLA-I$^+$ cancer cells.

Supplemental Figure 4. BCG infected cancer cells downregulate HLA-I and EpCAM molecules

Supplemental Figure 5. BCG downregulates HLA-I on U2OS Human osteosarcoma reporter cell line

Supplemental Figure 6. Methods for in situ immune profiling in a longitudinal cohort of paired bladder tumors pre- and post-BCG.

Supplemental Figure 7. Acquired resistance to BCG immunotherapy significantly induces upregulation of immune-checkpoint inhibitory receptors and tumor infiltrating CD8$^+$ lymphocytes.

Supplemental Figure 1. Ex vivo BCG stimulation of fresh human bladder tumors and cancer cell lines induces HLA-I downregulation on cancer cells.

(A) Bar plots showing the proportions of $CD45^+$ and $CD45^-$ cells in live cells among fresh bladder tumors based on flow cytometry analyses at baseline. BLCA: Bladder Cancer (n=12).

(B) Proportions (left panel) and mean fluorescence intensity (MFI, right panel) of HLA-I^+ in $CD45^+Epcam^-$ cells (n=12; One-way ANOVA with Tukey's post test).

(C) Relative proportions of tumor-infiltrating immune cells following in vitro BCG or IFNg stimulation of fresh human bladder tumors. Live leukocytes ($CD45^+$; top left panel; n=12; one-way ANOVA with Tukey's post test) and tumor-infiltrative T cells ($CD3^+$; top right panel) within those leukocytes (n=6; one-way ANOVA with Tukey's post test). Proportions of lymphoid cells ($CD4^+$, $CD8^+$, $FoxP3^+$, $CD56^+$ & $CD20^+$) and myeloid cells ($CD11b^+$) among live CD3+ or $CD45^+$ cells respectively, following in vitro stimulation with BCG or IFNg. Each dot represents one tumor (n=6; One-way ANOVA with Tukey's post test).

(D) Proportions of activated T cells (CD69 & PD1 on $CD3^+$ cells) and activated myeloid cells (HLA-DR^+ on $CD11b^+$ cells) following in vitro stimulation with BCG or IFNg. Each dot represents one tumor (n=6; One-way ANOVA with Tukey's post test).

(E) Flow cytometry histogram showing in vitro HLA-I downregulation in a subset of cells post-BCG exposure across 6 bladder cancer cell lines.

(F) Representative histogram showing in vitro β2-microgobulin downregulation in a subset of cells post-BCG exposure.

(G) β2-microgobulin MFI by flow-cytometry 24hrs upon in vitro BCG or IFNg exposure (n=3 conditions per cell line; one-way ANOVA with Tukey’s post test).

All data are presented as mean ± s.e.m.

Supplemental Figure 2. BCG induces EMT characteristics in HLA-I$^-$ cancer cells

(A) Phenotypic characteristics of HLA-I$^-$ cancer cells (in red) among cancer cell populations (in grey). Representative dot-plots for one cell line (5637).

(B) Representative imaging of untreated parental 5637 cancer cells (parental) and cell-sorted HLA-I$^-$ cells 24hrs after BCG exposure.

(C) Flow cytometry EpCAM expression 6 days after independent culture of HLA-I$^+$ and HLA-I$^-$ cancer cells in BCG-free medium shows sustained Epcam downregulation in RT4, 5637 and HT1376 cell lines.

(D) EMT score for urothelial cancer cell lines (n=8) based on selected gene expression profiles from the IO360 NanoString panel.

(E) MB49 and UPPL murine bladder cancer lines do not express HLA-I but can express it upon IFNg exposure (flow cytometry MFI of pan-HLA-I staining).

Supplemental Figure 3. BCG mostly induces inflammatory responses in HLA-I$^+$ cancer cells.

(A) Cytokine and chemokine levels (27-plex) in the supernatant of bladder cancer cells (n=3 cell lines, RT4, 5637, and UM-UC3) were measured separately in HLA-I$^+$ cells

24hrs after co-incubation with BCG, cell-sorting and independent culture in BCG-free medium for 72hrs (n=3 independent experiments in triplicate per cell line).

(B) Cytokine and chemokine levels (27-plex) in the supernatant of bladder cancer cells (n=3 cell lines, RT4, 5637, and UM-UC3) were measured separately in HLA-I$^-$ cells 24hrs after co-incubation with BCG, cell-sorting and independent culture in BCG-free medium for 72hrs (n=3 independent experiments in triplicate per cell line).

Supplemental Figure 4. BCG infected cancer cells downregulate HLA-I and EpCAM molecules

(A) Stimulation assay of cancer cells with TLR2, TLR4, TLR9, and combinations compared to BCG (one-way ANOVA with Tukey's post test).

(B) Percentage of cancer cells infected by calcein–labeled BCG and MFI calcein–labeled BCG among cell lines (n=3 cell lines; one-way ANOVA with Tukey's post test).

(C) Viability (MFI of calcein) of BCG upon different conditions: BCG not labelled with calcein, heat-killed BCG with calcein (80°C fo 60 min), UV-B exposed BCG with calcein (UV-B for 60 min), live BCG with calcein (one-way ANOVA with Tukey's post test).

Supplemental Figure 5. BCG downregulates HLA-I on U2OS Human osteosarcoma reporter cell line

(A) Illustration of the principles for autophagy measurements with LC3-GFP and LC3-RFP tandem U2OS Human osteosarcoma reporter cell lines. Briefly, autophagosomes fuse

with lysosomes to become autophagolysosomes. The LC3 protein is uniquely expressed within those intracytoplasmic vesicles. Autophagosomes are yellow in immunofluorescence upon co-expression of LC3-GFP and LC3-RFP proteins. Upon acidification of pH after fusion with lysosomes, the GFP fluorescence is no longer visible. However the RFP remains functional and the autophagolysosomes turn red. The autophagy flux increases when the number of red vesicles increases upon stimulation, whereas it is inhibited when more yellow vesicles (autophagosomes) accumulate in the cytoplasm. Figure adapted from *Lopez A, et al. Seeing is believing: methods to monitor vertebrate autophagy in vivo. Open Biol 2018;8:180106.* Of note, the GFP-LC3 cell line used in Figure 5E (without RFP) can only track autophagosomes. The GFP+RFP-LC3 tandem cell line depicted here and used for Figure 6F and 6G experiments can also assess the formation of autophagolysosomes.

(B) HLA-I negative cells in GFP-LC3 U2OS cells untreated (B, top and C, top left panel), and treated for 24hrs with BCG (B, bottom panel),

(C) HLA-I negative cells in GFP-LC3 U2OS cells treated for 48hrs with IFNg (C, top right, and bottom panels) treated for 24hrs with BCG (C, bottom panels) or not treated with BCG (C top right panel). One representative gate of three/four technical replicates is depicted.

(D) Proportions of HLA-I negative cells within live GFP-LC3 U2OS cells treated with or without BCG in presence or not of IFNg. Data from one experiment are showed as mean ± s.e.m. of three/four technical replicates (Kruskal-Wallis with Dunn’s post test).

Supplemental Figure 6. Methods for in situ immune profiling in a longitudinal cohort of paired bladder tumors pre- and post-BCG.

Supplemental Figure 7. Acquired resistance to BCG immunotherapy significantly induces upregulation of immune-checkpoint inhibitory receptors and tumor infiltrating CD8$^+$ lymphocytes.

(A) Expression of HLA-I on cancer cells pre- and post-BCG in the whole cohort (n=27).

(B) Volcano plot of differences of gene expression by NanoString profiling between BCG naïve and BCG resistant bladder tumors (*x* axis: Log2 fold-change (FC) of difference) and significance (*y* axis). Solid grey dots show the genes with significant difference (FDR<0.05).

(C) EMT score (RNA-Seq) and HLA-I expression (IHC) on cancer cells in a longitudinal series of paired tissue samples of patients acquiring BCG resistance and metastatic disease post-BCG (n=3).

(D) Representative images of CD3$^+$, CD8$^+$, CD68$^+$/CD163$^+$ and PD-L1 staining by immunohistochemistry in BCG resistant tumors are shown.

(E) Immune profiling by immunohistochemistry in tumor samples pre- and post-BCG in the whole cohort (n=27). Density of CD3$^+$(%) cells, CD8$^+$(%) cells, CD8$^+$ in CD3$^+$ cells (%), CD68$^+$ (%) cells, CD163$^+$ (%) cells and PD-L1 expression on immune cells (%) are shown. Two-tailed paired t-tests.

(F) Heat map illustrating the level of NK- and gamma-delta T cells related gene expression in HLA-I$^-$ and HLA-I$^+$ bladder tumors post-BCG (Limma *P* value). In situ gene expression analysis by RNA-Seq.

(G) Relative abundance of NK cells in HLA-I$^-$ and HLA-I$^+$ bladder tumors post-BCG (unpaired two-tailed t test). Estimation of the abundance of the NK and gd T cells population using the computational method MCP-counter (58).

All data are presented as mean ± s.e.m.

Supplemental Figure 1

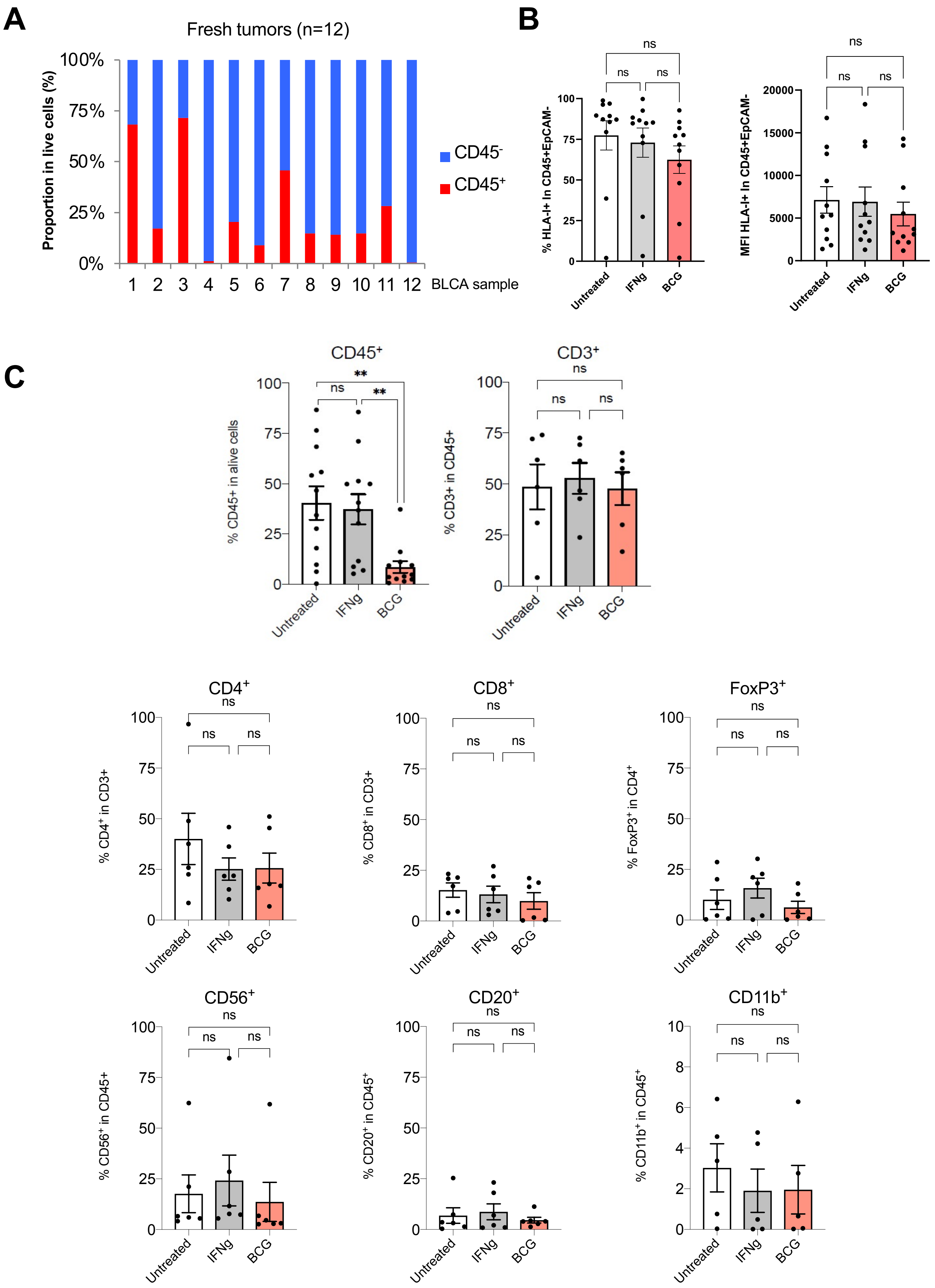

Supplemental Figure 1

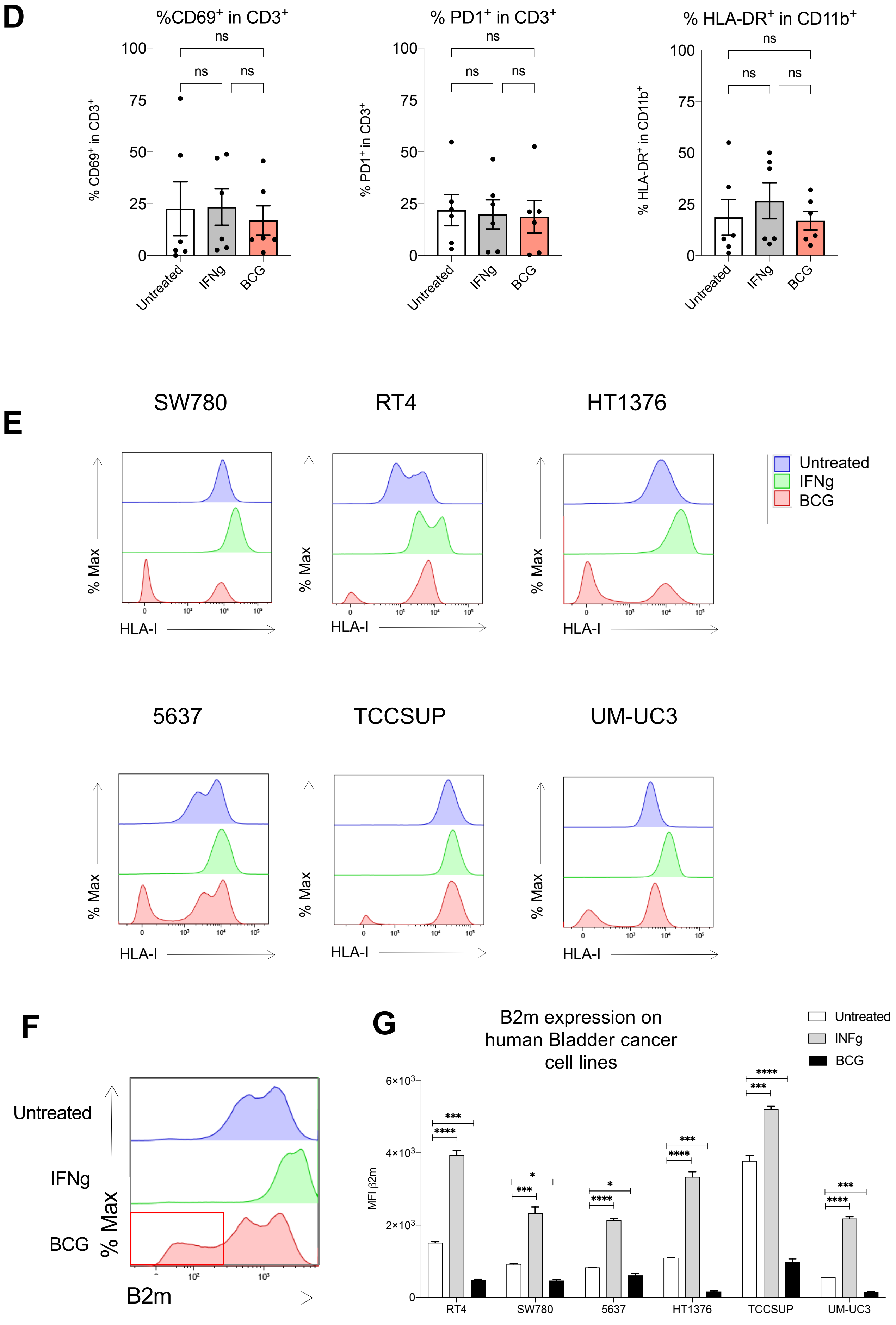

Supplemental Figure 2

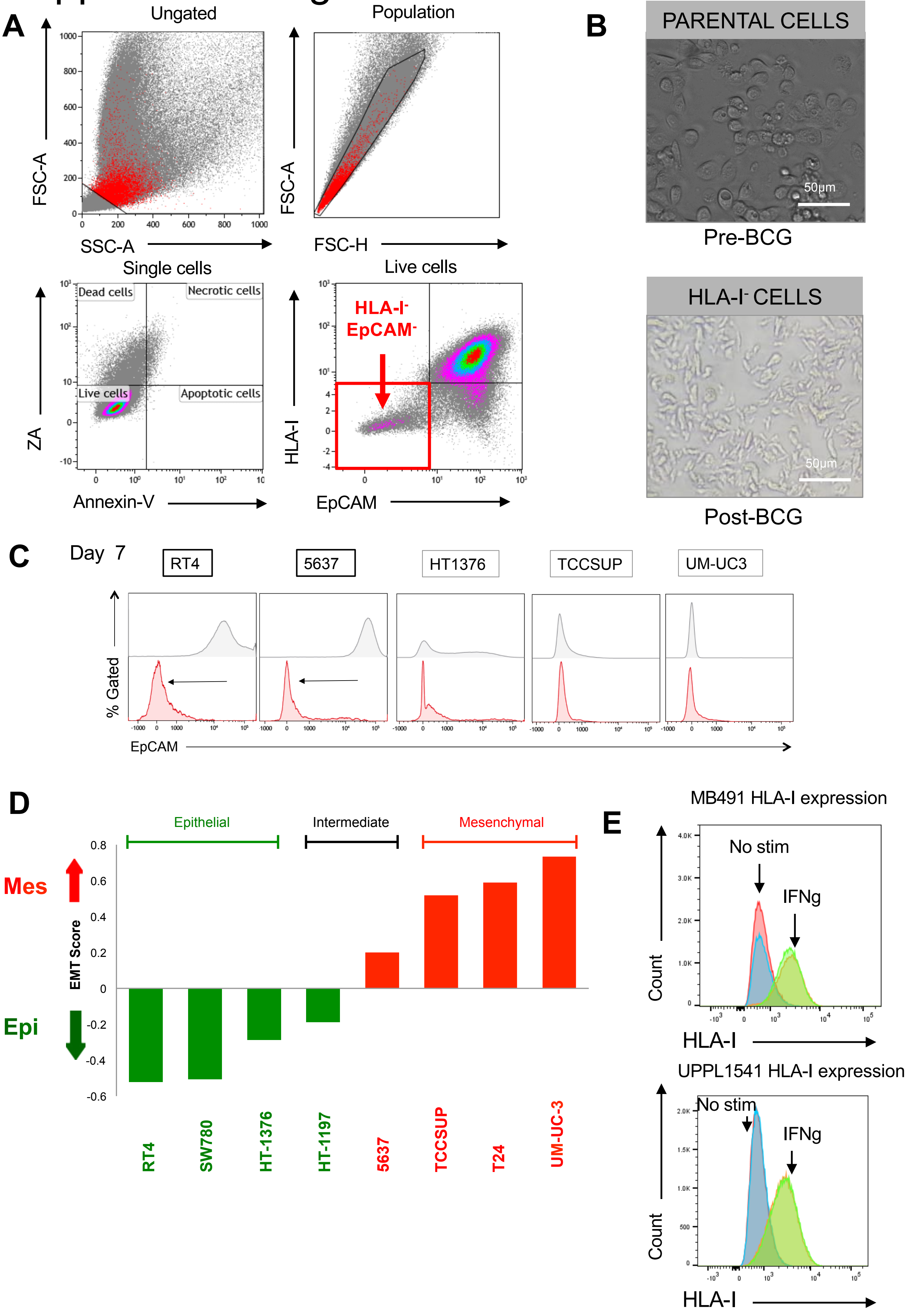

Supplemental Figure 3

A

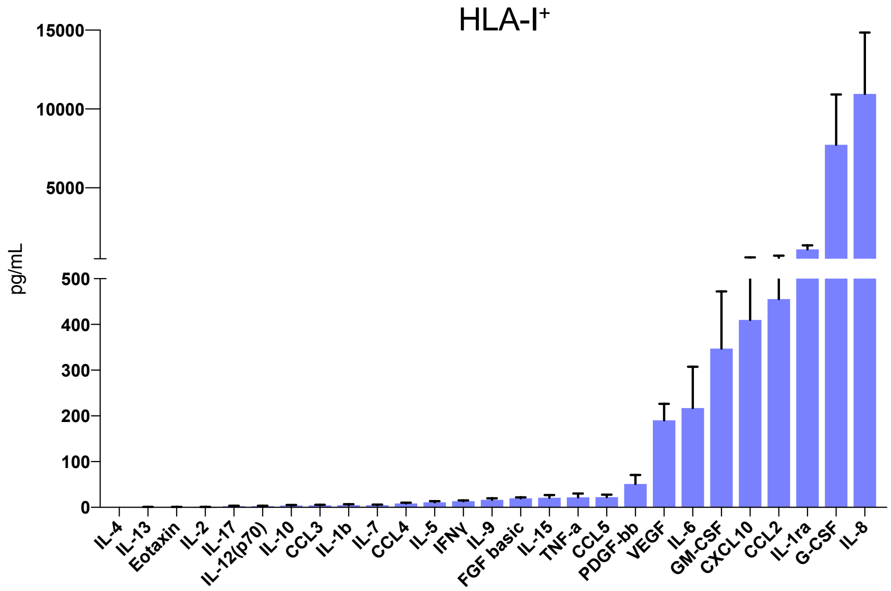

B

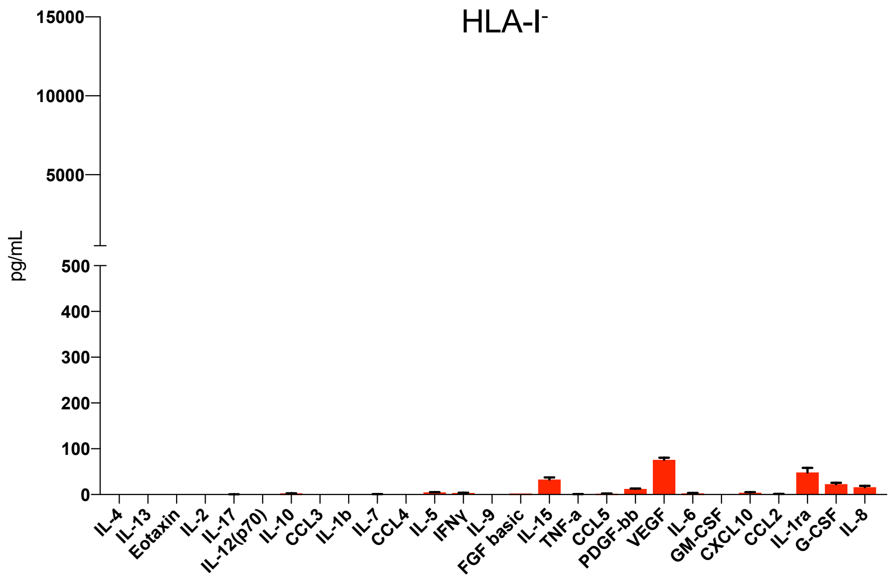

Supplemental Figure 4

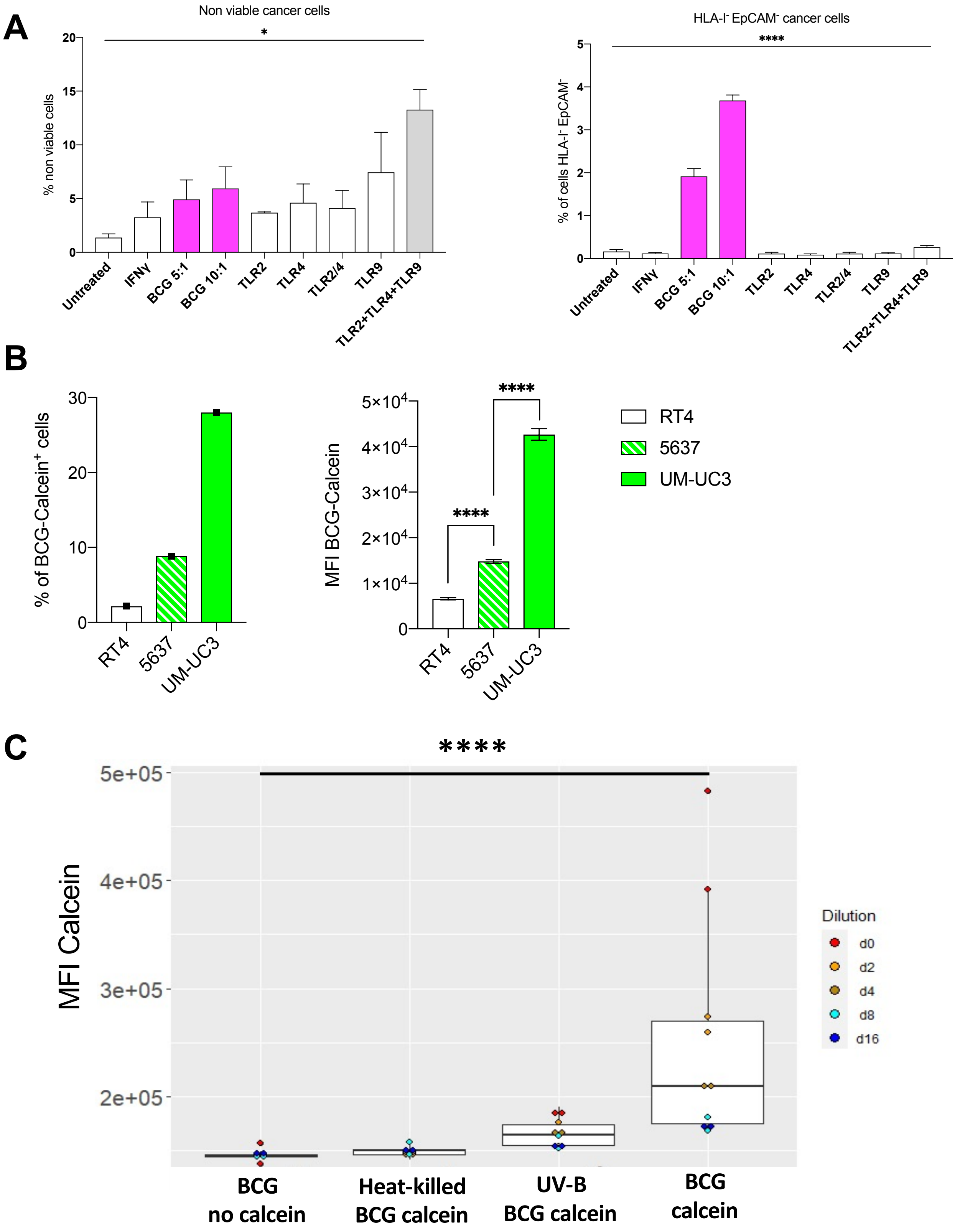

Supplemental Figure 5

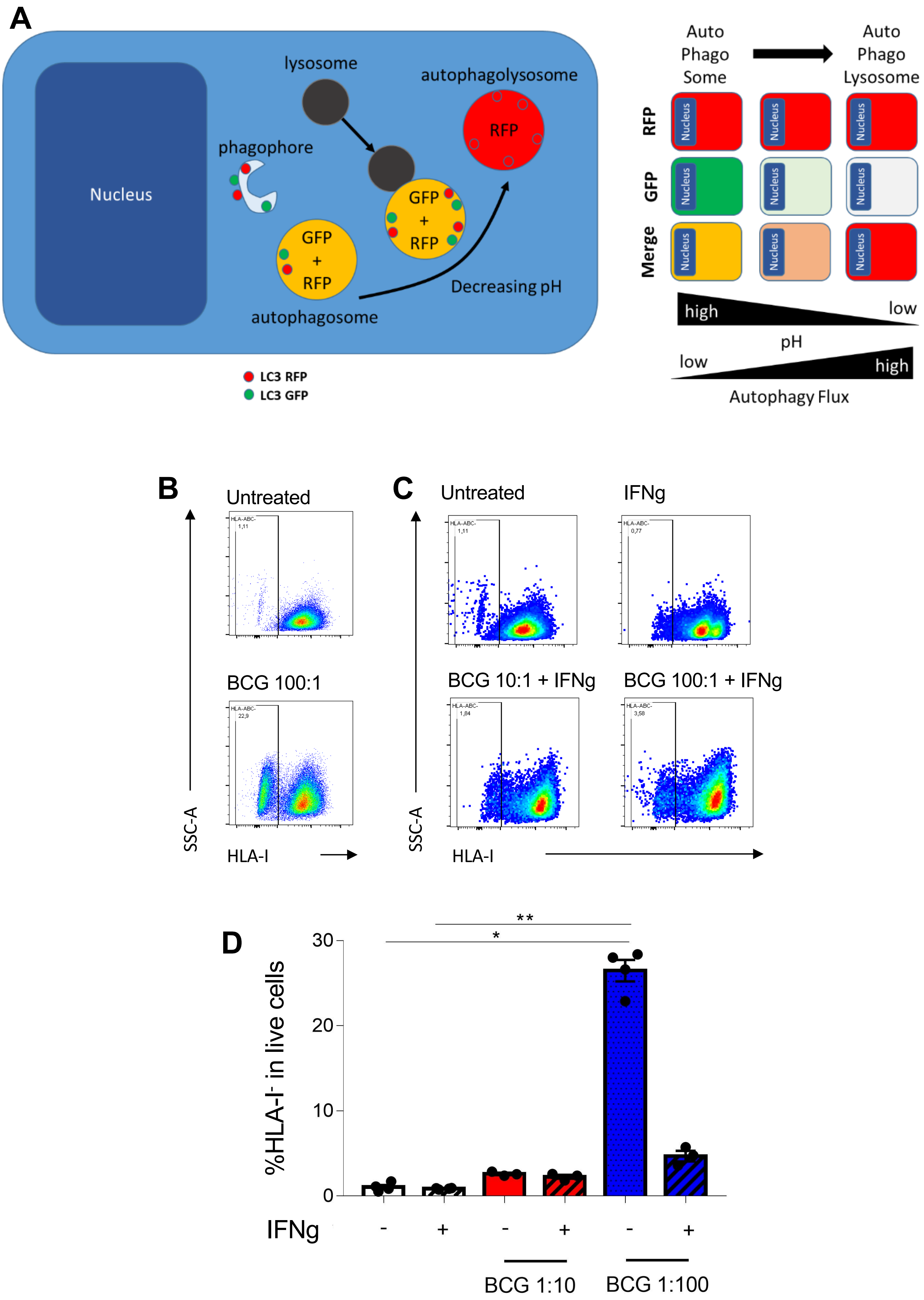

Supplemental Figure 6

A Tumor blocks selection of paired bladder tumors

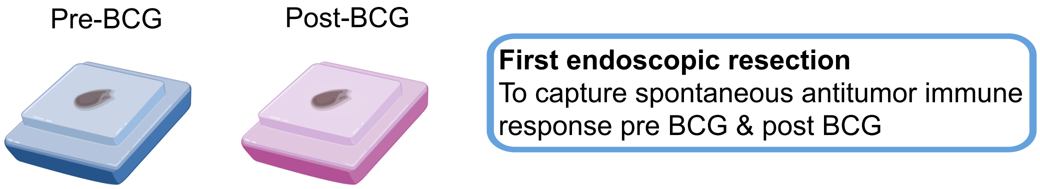

B Tumor areas selection

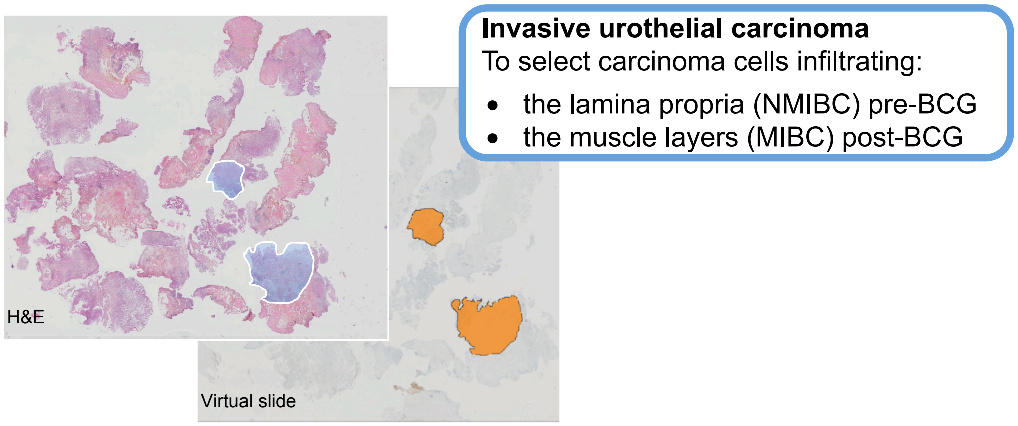

C Combined analysis of the same tumor areas with gene and protein expression

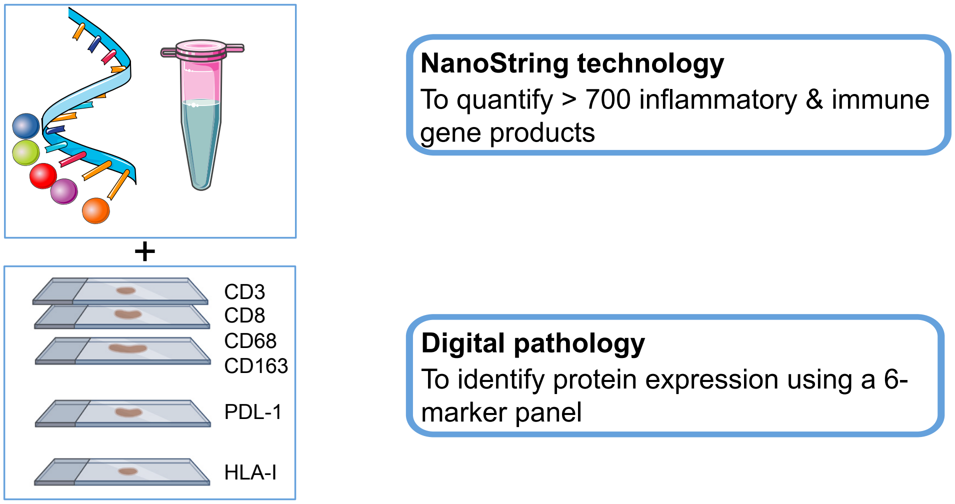

Supplemental Figure 7

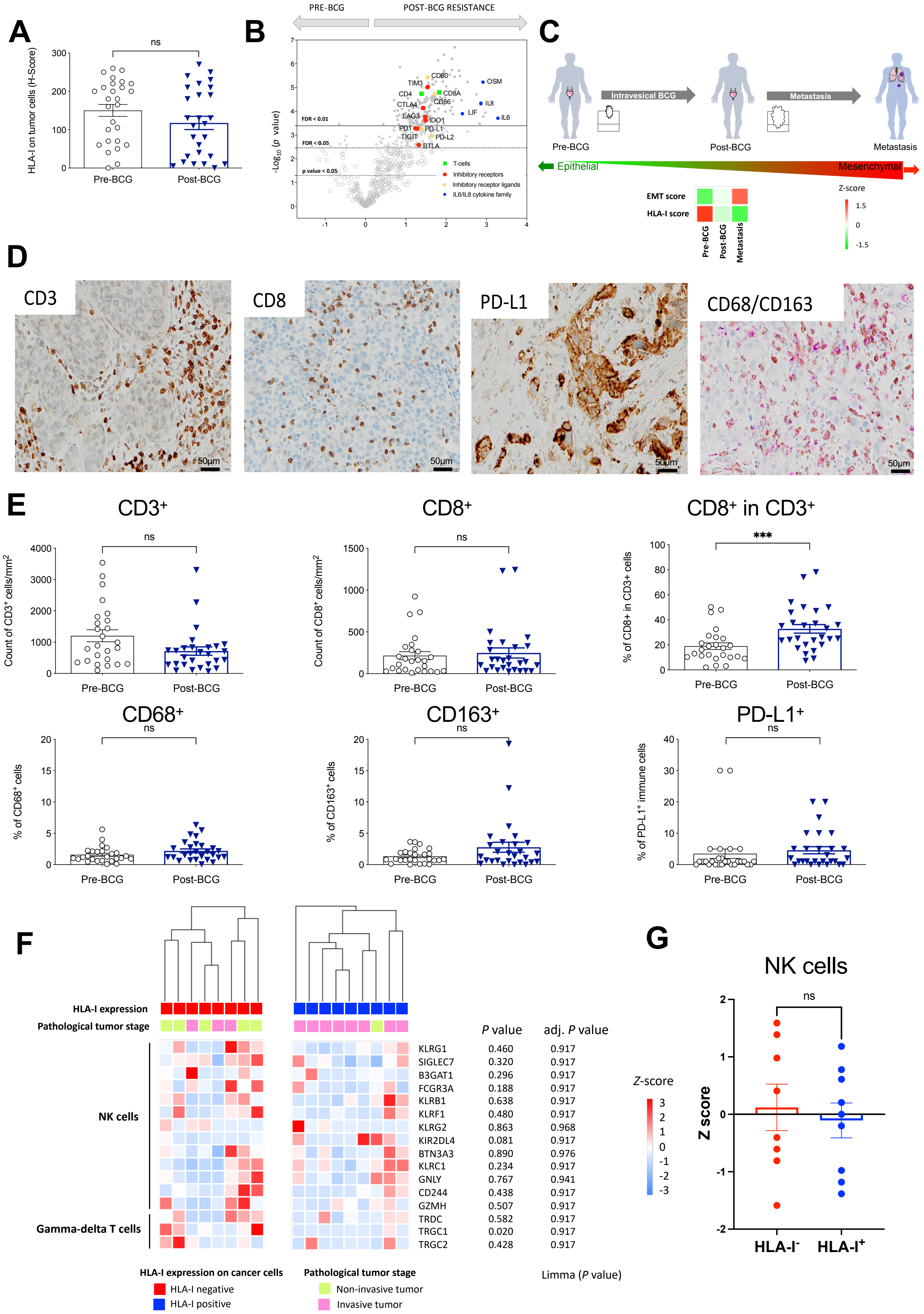

Supplemental Tables

Supplemental Table 1. Clinical features of fresh bladder tumor donors (n=12).

Supplemental Table 2. List of genes selected from NanoString IO360 panel to calculate the EMT score for each bladder cancer cell line (adapted from *(17)* T.Z. Tan et al.)

Supplemental Table 3. Clinical characteristics of the RNA-seq cohort of BCG-treated bladder cancer patients (n=14).

Supplemental Table 4. Autophagy pathway gene set.

Supplemental Table 5. Clinical characteristics of patients from our cohort of bladder tumors with BCG acquired resistance (n=27)

Supplemental Table 6. NK and gdT cell gene sets

Supplemental Table 1: Clinical features of fresh bladder tumors donors (n=12)

SAMPLE ID	Age	Gender	Prior therapy	Surgery	Grade	CIS[a]	Stage	Pathology
BLCA 1	60	M	None	Cystectomy	High	No	Ta	Urothelial carcinoma
BLCA 2	78	M	None	Cystectomy	High	Yes	Ta	Urothelial carcinoma
BLCA 3	73	M	None	Cystectomy	High	Yes	T3b	Urothelial carcinoma
BLCA 4	74	M	None	Cystectomy	High	Yes	T3b	Urothelial carcinoma
BLCA 5	69	M	BCG	Cystectomy	High	Yes	T3b	Micropapillary variant
BLCA 6	81	F	BCG	Cystectomy	High	Yes	T3b	Urothelial carcinoma
BLCA 7	89	M	None	Cystectomy	High	Yes	T3b	Urothelial carcinoma
BLCA 8	80	M	None	Cystectomy	High	Yes	T3b	Urothelial carcinoma
BLCA 9	86	M	None	Cystectomy	High	Yes	T3b	Urothelial carcinoma
BLCA 10	82	M	None	Cystectomy	High	Yes	T3b	Urothelial carcinoma
BLCA 11	70	F	Chemotherapy	Cystectomy	High	No	T4a	Urothelial carcinoma
BLCA 12	65	M	None	Cystectomy	High	No	Ta	Urothelial carcinoma

[a] Concomittant carcinoma *in situ* (CIS)
BLCA : bladder cancer

Supplemental Table 2 List of genes selected from NanoString IO360 panel to calculate the EMT score for each bladder cancer cell line

Index	Gene symbol	Gene title	GO molecular function	Category
1	*CDH1*	Cadherin 1, type 1, E-cadherin (epithelial)	Calcium ion binding	Epi
2	*AREG*	Amphiregulin	Growth factor activity	Epi
3	*AXL*	Tyrosine-protein kinase receptor UFO	Non-membrane spanning protein tyrosine kinase activity; transmembrane receptor protein kinase, growth factor activity	Mes
4	*CBLC*	Signal transduction protein CBL-C	Ligase activity	Epi
5	*DTX4*	Protein deltex-4	Transcription factor activity	Epi
6	*EPCAM*	Epithelial cell adhesion molecule	Receptor activity	Epi
7	*ERBB2*	Receptor tyrosine-protein kinase erbB-2	Non-membrane spanning protein tyrosine kinase activity ; growth factor activity	Epi
8	*FBP1*	Fructose-1,6-bisphosphatase 1	Protein binding	Epi
9	*HES1*	Transcription factor HES-1	Transcription factor activity	Epi
10	*IL1RN*	Interleukin-1 receptor antagonist protein	Hematopoietin/interferon-class (D200-domain) cytokine receptor binding	Epi
11	*LOXL2*	Lysyl oxidase homolog 2	Oxidoreductase activity;serine-type peptidase activity;receptor activity	Mes
12	*OAS1*	2'-5'-oligoadenylate synthase 1	Nucleotidyltransferase activity;nucleic acid binding	Epi
13	*TNFSF13*	Tumour necrosis factor ligand superfamily member 13	---	Epi
14	*ZEB1*	Zinc finger E-box-binding homeobox 1	---	Mes
15	*CDH2*	Cadherin-2	G-protein coupled receptor activity;calcium ion binding	Mes

Supplemental Table 3. Clinical characteristics of the RNA-seq cohort of BCG-treated bladder cancer patients (n=14)

		RNA-seq cohort n=14 (100%)	Post BCG HLA$^-$ n=8 (57%)	Post BCG HLA-I$^+$ n=6 (43%)	p value[a]
**Age, year**	Median	73.5	73.5	73	0.33
	Range	46-85	46-85	67-82	
**Gender**	Female	0 (0)	0 (0)	0 (0)	1
	Male	14 (100)	8 (100)	6 (100)	
**Smoking status**	Never	2 (14)	1 (12.5)	1 (17)	0.35
	Former	7 (50)	4 (50)	3 (50)	
	Current	1 (7)	1 (12.5)	0 (0)	
	Unknown	4 (29)	2 (25)	2 (33)	
**Primary therapy[c]**	Intravesical BCG	14 (100)	8(100)	6 (100)	1
	Other	0 (0)	0 (0)	0 (0)	
**Pre BCG tumor staging**	T1N0M0	14 (100)	8 (100)	6 (100)	1
	CIS[b]	7 (50)	2 (25)	5 (83)	
**Progression to MIBC**	Yes	9 (64)	4 (50)	5 (83)	0.23
	No	5 (36)	4 (50)	1 (17)	
**Post BCG tumor staging**	<T2N0M0	6 (43)	5 (62.5)	1 (17)	0.1
	≥T2N0M0	8 (57)	3 (37.5)	5 (83)	
**Radical cystectomy**	Yes	10 (71)	5 (62.5)	5 (83)	0.1
	No	4 (29)	3 (37.5)	1 (17)	
**Perioperative chemotherapy**	Yes	2 (14)	1 (12.5)	1 (17)	0.84
	No	12 (86)	7 (87.5)	5 (83)	

Supplemental Table 4. Autophagy pathway gene set

Gene symbol	Gene title	Function
ATG3	Autophagy Related 3	E2 ubiquitin-like-conjugating enzyme
ATG5	Autophagy Related 5	ATG12-ATG5 is an E3 ubiquitin-like-conjugating enzyme
ATG7	Autophagy Related 7	E1 ubiquitin-like-conjugating enzyme
ATG12	Autophagy Related 12	ATG12-ATG5 is an E3 ubiquitin-like-conjugating enzyme
ATG13	Autophagy Related 13	Required for the autophagosome formation
ATG14	Autophagy Related 14	Involved in the autophagosome formation
ATG16L1	Autophagy Related 16 Like 1	Activates the elongation of the autophagosomal membrane
BECN1	Beclin 1	Core subunit of the PI3K complex
GABARAP	GABA Type A Receptor-Associated Protein	Required for the autophagosome maturation
MAP1LC3A	Microtubule Associated Protein 1 Light Chain 3 Alpha	Involved in the autophagosome formation
PIK3C3	Phosphatidylinositol 3-Kinase Catalytic Subunit Type 3	Catalytic subunit of the PI3K complex, PI3KC3-C1 and C2 are involved in the autophagosome formation and maturation respectively
0PIK3R4	Phosphoinositide-3-Kinase Regulatory Subunit 4	Regulatory subunit of the PI3K complex
RAB7A	RAB7A, Member RAS Oncogene Family	Small GTPase, regulator of the autophagosome maturation
RAB7B	RAB7B, Member RAS Oncogene Family	Small GTPase, inhibitor of autophagy
RB1CC1	RB1 Inducible Coiled-Coil 1	Regulates the autophagosome formation
SNAP29	Synaptosome Associated Protein 29	Soluble N-ethylmaleimide-sensitive factor-attachment protein receptor (SNARE), controls the fusion of the autophagosome with the lysosome
STX17	Syntaxin 17	SNARE, controls the fusion of the autophagosome with the lysosome
ULK1	Unc-51 Like Autophagy Activating Kinase 1	Regulates the autophagophore formation
ULK2	Unc-51 Like Autophagy Activating Kinase 2	Regulates the autophagophore formation
VAMP8	Vesicle Associated Membrane Protein 8	SNARE, controls the fusion of the autophagosome with the lysosome

Supplemental Table 5. Clinical characteristics of patients from our cohort of bladder tumors with BCG acquired resistance (n=27)

		Total cohort n=27 (100%)	Outcome Metastases n=11 (41%)	Outcome No metastasis n=16 (59%)	p value[a]
Age, year	Median	73.5	73.5	73.5	0.69
	Range	46-91	46-88.5	56-91	
Gender	Female	4 (15)	3 (27)	1 (6)	0.14
	Male	23 (85)	8 (73)	15 (94)	
Smoking status	Never	9 (33)	5 (45)	4 (25)	0.17
	Former	10 (37)	5 (45)	5 (31)	
	Current	6 (22)	1 (10)	5 (31)	
	Unknown	2 (7)	0 (0)	2 (12)	
Primary therapy[c]	Intravesical BCG	27 (100)	11 (100)	16 (100)	1
	Other	0 (0)	0 (0)	0 (0)	
Pre BCG tumor staging	T1N0M0	27 (100)	11 (100)	16 (100)	1
	CIS[b]	16 (59)	5 (45)	11 (69)	
Progression to MIBC	Yes	27 (100)	11 (100)	16 (100)	1
	No	0 (0)	0 (0)	0 (0)	
Post BCG tumor staging	T2N0M0	16 (59)	7 (64)	9 (56)	0.50
	>T2N0M0	11 (41)	4 (36)	7 (44)	
Radical cystectomy	Yes	20 (74)	7 (64)	13 (81)	0.33
	No	7 (26)	4 (36)	3 (19)	
Perioperative chemotherapy	Yes	4 (20)	1 (14)	3 (23)	0.66
	No	(26)	6 (86)	10 (77)	

[a] *P* value < 0.05 are significant.
[b] Concomitant carcinoma in situ (CIS) at diagnosis.
[c] None of the patients received BCG prior to collection of the analyzed samples.

Supplemental Table 6. NK and gdT cells gene sets

Gene symbol	Gene title	Function
KLRG1	killer cell lectin like receptor G1	Inhibitor of NK cytotoxicity
SIGLEC7	sialic acid binding Ig like lectin 7	Inhibitor of NK cytotoxicity
B3GAT1	beta-1,3-glucuronyltransferase 1	Biosynthesis of the HNK-1 epitope on glycoproteins
FCGR3A	Fc fragment of IgG receptor IIIa	Receptor for the Fc fragment of immunoglobulin (Ig)G
KLRB1	killer cell lectin like receptor B1	Inhibitor of NK cytotoxicity
KLRF1	killer cell lectin like receptor F1	Activator of NK cytotoxicity
KLRG2	killer cell lectin like receptor G2	Receptor with a C-type lectin domain
KIR2DL4	killer cell immunoglobulin like receptor, two Ig domains and long cytoplasmic tail 4	Activator of NK cytokine and chemokine secretion
BTN3A3	butyrophilin subfamily 3 member A3	receptor glycoproteins of Ig superfamily
KLRC1	killer cell lectin like receptor C1	Inhibitor of NK cytotoxicity
GNLY	granulysin	Activator of NK cytotoxicity
CD244	cluster of differentiation 244	Activator of NK cytotoxicity
GZMH	granzyme H	Activator of NK cytotoxicity
TRDC	T cell receptor delta constant	Constant region of the TCR delta chain
TRGC1	T cell receptor gamma constant 1	Constant region of the TCR gamma chain
TRGC2	T cell receptor gamma constant 2	Constant region of the TCR gamma chain
